# Double Deletion of EP402R and EP153R in the Attenuated Lv17/WB/Rie1 African Swine Fever Virus (ASFV) Enhances Safety, Provides DIVA Compatibility, and Confers Complete Protection Against a Genotype II Virulent Strain

**DOI:** 10.3390/vaccines12121406

**Published:** 2024-12-13

**Authors:** Carmina Gallardo, István Mészáros, Alejandro Soler, Jovita Fernandez-Pinero, Erwin van den Born, Alicia Simón, Nadia Casado, Raquel Nieto, Covadonga Perez, Irene Aldea, Vicente Lopez-Chavarrias, Eszter Göltl, Ferenc Olasz, Tibor Magyar, Zoltán Zádori, José Manuel Sánchez-Vizcaíno, Marisa Arias

**Affiliations:** 1 European Union Reference Laboratory for African Swine Fever (EURL), Centro de Investigación en Sanidad Animal (CISA), Instituto Nacional de Investigación y Tecnología Agraria y Alimentaria (INIA), Consejo Superior de Investigaciones Científicas (CSIC), Valdeolmos, 28130 Madrid, Spain; alejandro.soler@inia.csic.es (A.S.); fpinero@inia.csic.es (J.F.-P.); alicia.simon@inia.csic.es (A.S.); nadia.casado@inia.csic.es (N.C.); nieto.raquel@inia.csic.es (R.N.); cperez@inia.csic.es (C.P.); arias@inia.csic.es (M.A.); 2 HUN-REN Veterinary Medical Research Institute, Hungária krt. 21, 1143 Budapest, Hungary; meszaros.istvan@vmri.hun-ren.hu (I.M.); goltl.eszter@vmri.hun-ren.hu (E.G.); olasz.ferenc@vmri.hun-ren.hu (F.O.); magyar.tibor@vmri.hun-ren.hu (T.M.); zadori.zoltan@vmri.hun-ren.hu (Z.Z.); 3 MSD Animal Health, 5831 AN Boxmeer, The Netherlands; 4 Centro de Investigación en Sanidad Animal (CISA), Instituto Nacional de Investigación y Tecnología Agraria y Alimentaria (INIA), Consejo Superior de Investigaciones Científicas (CSIC), Valdeolmos, 28130 Madrid, Spain; irene.aldea@inia.csic.es (I.A.); vicente.lopez@inia.csic.es (V.L.-C.); 5 VISAVET Health Surveillance Center, Complutense University of Madrid, 28040 Madrid, Spain; jmvizcaino@ucm.es

**Keywords:** ASFV, attenuated virus, Lv17/WB/Rie1, vaccine, EP402R, EP153R, recombinant virus pathology

## Abstract

**Background/Objectives:** African swine fever virus (ASFV) is a devastating disease affecting domestic and wild suids and causing significant economic losses in the global pig industry. Attenuated modified live virus (MLV) vaccines are the most promising approaches for vaccine development. This study aimed to evaluate the safety and efficacy of four recombinant ASFV genotype II strains, derived from the non-hemadsorbing (non-HAD) attenuated isolate Lv17/WB/Rie1, through the single or simultaneous deletion of virulence-associated genes. **Methods:** Recombinant viruses were engineered by deleting the *UK*, *EP402R*, and *EP153R* genes, either individually or in combination. Four recombinant strains were evaluated for safety and efficacy in domestic pigs vaccinated intramuscularly with 10^2^ TCID₅₀. Clinical signs, viremia, virus shedding, and antibody responses were monitored. Protection efficacy was assessed by challenging vaccinated pigs with the virulent genotype II Armenia07 strain. Additionally, a reversion-to-virulence study involving an overdose of the vaccine candidate was conducted to evaluate its stability through serial immunizations. **Results:** Deletion of the *UK* gene alone increased virulence, whereas the double deletion of *EP402R* and *EP153R* (Lv17/WB/Rie1-ΔCD) significantly enhanced safety while maintaining full protective efficacy. Vaccinated pigs exhibited reduced viremia, no virus shedding, and robust virus-specific antibody responses, achieving complete protection against Armenia07. The reversion-to-virulence study revealed potential but limited pathogenicity after multiple passages, indicating areas for improvement in vaccine stability. **Conclusions:** The Lv17/WB/Rie1-ΔCD strain demonstrates excellent safety and efficacy, along with potential DIVA (differentiating infected from vaccinated animals) compatibility, positioning it as a strong candidate for an ASFV MLV vaccine. Further research is needed to refine the vaccine and address the potential risks of reversion to virulence.

## 1. Introduction

African swine fever (ASF) is a devastating hemorrhagic disease of domestic pigs and wild suids caused by a large double-stranded DNA virus, African swine fever virus (ASFV). ASFV is the only member of the Asfarviridae family and only members of the Suidae family are susceptible to ASFV, which has no zoonotic potential [1,2].

Virulent strains of ASFV can kill domestic pigs within about 5–14 days of infection, with a mortality rate approaching 100% [3]. Vaccination is considered the most efficient tool for the control of animal infectious diseases. However, a universal global vaccine is not available for ASF so far. In the past, some countries reached eradication by using several strategies that included widespread culling and improvements in biosecurity on farms [4]. ASF has evolved over the past decade from a localized disease in Sub-Saharan Africa to a major global challenge for pig production. The spread of genotype II, which began with its introduction in Georgia in 2007, has since extended through the Russian Federation and Eastern Europe, reaching the European Union (EU) in 2014 [5]. Since then, 23 countries have declared the disease in Europe, mainly affecting European wild boar, with 62,943 cases (83.9%), compared to 12,061 outbreaks (16.1%) in domestic pigs (EU Animal Notification System (ADNS), updated 04/09/2024). Its rapid spread in the north of mainland Italy, approaching around 60 km from the border with Switzerland in 2024, highlights an extremely disconcerting pattern of continued spread of ASFV in Europe [6]. The first ASF outbreak in East Asia was confirmed in pigs in China in August 2018, from where it rapidly spread across the country from north to south and reached 15 other Asian countries and the Pacific (Papua New Guinea) in just 3 years. As of June 2024, up to 20 countries in the region have had occurrences of the disease, with Singapore (2023) and Bangladesh (2023) being the last to be affected. It has led to huge economic losses in many Asian countries [7]. In total, since January 2022, ASF has been reported as present in 59 countries in five different world regions, spreading through Europe, Asia, and the Pacific and reappearing in America (Dominican Republic and Haiti) in 2020, 40 years after its first incursion into this continent [8].

It is clear from the current situation that available control measures have failed to eliminate the disease in most countries affected by ASF. Pork is one of the main sources of animal protein and represents more than 35% of global meat consumption. Hence, this disease represents a serious problem for food security worldwide, as well as for biodiversity and the balance of ecosystems, with wild boar being one of the most affected species, particularly in the EU. Therefore, developing a safe and effective vaccine, along with implementing targeted vaccination programs, is now more crucial than ever as an additional tool for control, tailored to specific scenarios and economic activities. However, the ASF virus poses significant challenges due to its complexity, encoding over 160 different polypeptides, many of which are specialized in evading various aspects of the immune system [9]. This, combined with the variability among virus isolates, has made controlling the virus difficult for decades. Additionally, the infection of immune defense cells, specifically monocytes and macrophages, and a limited understanding of the mechanisms behind immune evasion continue to create major gaps in our knowledge of the virus’s pathogenesis and virus-induced immunity. Nevertheless, evidence suggests that a protective immune response involves both cellular and serological immunity [10], with some level of protection attributed to antibody-mediated immunity.

The research efforts in vaccine development have been intensified in recent years, with attenuated modified live virus (MLVs) vaccines based on the deletion of virulence-associated genes being the most promising as they confer complete protection against homologous lethal field strains [11,12,13]. The fact that two MLVs, ASFV-G-ΔI177L and ASFV-G-∆MGF [14,15,16,17], are the first commercial vaccines against ASFV in Vietnam raises hopes that a licensed product based on such vaccines is on the horizon at the global level. However, there are still significant safety concerns. The use of MLVs derived from virulent virus strains raises concerns about the potential for vaccine virus persistence, reversion to a virulent form, or recombination with wild-type strains, leading to the emergence of new variants [18]. Efforts to enhance MLV safety are essential to mitigate these risks effectively. The development of deletion mutants based on naturally attenuated virus isolates could be the most promising option to reduce concerns about reversion to virulence, control the spread of ASFV, and reduce the risk of its devastating consequences throughout the world [12].

The naturally attenuated field strain Lv17/WB/Rie1 (genotype II), isolated in 2017 from a hunted wild boar in Latvia, has been shown to produce mild clinical signs with transitory fever and obtained good results as regards protective immunity against virulent isolates in both wild boar and domestic pigs [19,20]. However, the immunoprotective effect and especially the safety of the attenuated strain vary depending on the immune status of the host, the dose, and the route of administration. The fact is that animals immunized with the naturally attenuated Lv17/WB/Rie1 strain present variable side effects such as fever, skin damage, and joint inflammation, making it difficult to use this strain as a vaccine against ASFV. Post-vaccination reactions may lead to the development of chronic ASF [20]. Moreover, the requirement of DIVA (differentiating infected from vaccinated animals) characteristics for authorization of a vaccine entails the genetic manipulation of the Lv17/WB/Rie1 strain as well.

In this study, we deleted three genes, alone or in combination, from the field-attenuated and non-hemadsorbing (non-HAD) genotype II ASFV Lv17/WB/Rie1 to produce four different recombinant viruses. The identified ASFV viral factors related to hemadsorption (HAD) were the products encoded by the genes *EP153R* [21] and *EP402R* (encoding the CD2v protein) [22]. The HAD phenotype facilitates virus spread and persistence in infected animals by maintaining a large proportion of ASFV in the red blood cell fraction of the blood [22]. We previously demonstrated that the *EP402R* gene in the non-HAD ASFV Lv17/WB/Rie1 has a single adenosine deletion at position 73,763 of the genome (patent WO 2020/049194 A1) that affects the N-terminal extracellular domain of CD2v protein [20]. The N-terminal extracellular domain of the CD2v protein, called the “ligand-binding domain” (formed by amino acids 1-203 of the *EP402R* gene), is essential for efficient gene expression. Mutations of one or more nucleotides in the ligand-binding domain can alter the folding of the CD2v protein. However, depending on the mutation, altered folding may mean that the protein folds differently and is expressed more slowly but correctly, leading to the HAD phenotype [23,24]. Therefore, to avoid reversion to a HAD phenotype and thus reversion to a more virulent phenotype, both genes involved in HAD, alone or in combination, were deleted from Lv17/WB/Rie1. Additionally, CD2v (*EP402R*) and C-type lectin protein (*EP153R*) mediate the serological specificity of HAD inhibition (HAI) and could potentially contain CD8+ T cell determinants capable of activating the cellular and humoral immune response against ASFV [25,26,27,28]. This fact could provide a solid basis for the development of DIVA diagnostic methods accompanying the developed vaccines. Additionally, we decided to extend our assessment of increasing ASFV Lv17/WB/Rie1 attenuation by deleting another virus gene involved in virulence, the *UK* gene [29,30].

In this study, we present the construction of four recombinant versions of the Lv17/WB/Rie1 strain lacking the above mentioned genes in different combinations: Lv17/WB/Rie1-ΔUK, Lv17/WB/Rie1-ΔEP153R, Lv17/WB/Rie1-ΔCD (lacking both the *EP402R* and *EP153R* genes), and Lv17/WB/Rie1-ΔCD/ΔUK (lacking the *EP402R*, *EP153R*, and *UK* virus genes). We conducted an in-depth analysis of the results obtained from the “in vivo” experimental immunization of domestic pigs with these four recombinant viruses, followed by a challenge with the highly virulent genotype II ASFV strain Armenia07. Additionally, we include an in vivo experiment assessing the reversion to virulence of the most promising prototype vaccine.

## 2. Materials and Methods

### 2.1. Cells and Viruses

Porcine peripheral blood monocytes (PBMs) were derived from Large White outbred pigs typically 8–12 weeks old. PBMs were prepared from un-clotted fresh blood using a mechanical defibrinator as described in the WOAH Manual [31]. After three-day culture, PBMs were infected with Lv17/WB/Rie1 ASFV at a multiplicity of infection (MOI) of 0.1, with the natural attenuated and non-HAD genotype II isolate used as a parental strain (patent WO 2020/049194 A1). Cells were incubated at 37 °C in 5% CO_2_ for 7 days, after which the culture medium containing the extracellular virus was collected and centrifuged at a low speed to remove cellular debris and then at 15,000× *g*, 4 °C, to sediment the virus [32]. The titration of the non-HAD ASFV Lv17/WB/Rie1 stock was performed on Cos7 [32] to monitor the end-point dilution, and viral titer was determined as the amount of the virus causing a cytopathic effect using immunoperoxidase staining in 50% of infected cultures (TCID_50_/mL) [33]. The highly virulent hemadsorbing (HAD) genotype II ASFV Armenia07 (Arm07) isolate, which shares 99.99% homology with the reference Georgia 2007 ASFV strain (GenBank accession number FR682468.2), was used as the challenge virus [33,34]. The virus was propagated in PBMs and the viral titer was defined as the amount of virus causing HAD in 50% of infected cultures (HAD_50_/mL) [32].

The Cos7 cell culture used for titration of the viruses was originally obtained from the American Type Culture Collection (ATCC CRL-1651) and it was grown in Dulbecco’s modified Eagle Medium (DMEM) (Capricorn, Ebsdorfergrund, Germany) supplemented with 2mM L-glutamine, 100U of gentamicin per ml (Sigma-Aldrich, Saint Louis, MO, USA), 1% Na Pyruvate (Thermo Scientific, Waltham, MA, USA), and 1% non-essential amino acids (Thermo Scientific, Waltham, MA, USA). Cells were cultured at 37 °C in medium supplemented with 10% FBS.

The porcine alveolar macrophages (PAMs) used to grow the recombinant viruses were prepared according to the WOAH Manual [31] and were stored in RPMI-1640 medium (Thermo Scientific, Waltham, MA, USA) supplemented with 30% fetal bovine serum (Sigma-Aldrich, Saint Louis, MO, USA) and 10% DMSO at −72 °C. PAMs were cultured in RPMI-1640 containing 10% (*v*/*v*) heat-inactivated fetal bovine serum (FBS) (Thermo Scientific, Waltham, MA, USA) plus 1X antibiotic/antimycotic solution (Thermo Scientific, Waltham, MA, USA) and 2 mM L-glutamine (Sigma-Aldrich, Saint Louis, MO, USA). Cells were incubated at 37 °C in 5% CO_2_ for 24 h before the infection.

The MS-stable monkey kidney cell line (ECACC, 91070510) was used for the preparation of the ASFV MS-adapted E70 isolate (E70 MS 48)-coated 96-well plates. These plates were then used as the antigen in the indirect immunoperoxidase test (IPT) [31].

### 2.2. Construction of the Recombinant ASFVs Lv17/WB/Rie1-∆EP153R, Lv17/WB/Rie1-∆UK, Lv17/WB/Rie1-ΔCD and Lv17/WB/Rie1-ΔCD/ΔUK

All four mutant viruses were manufactured as described earlier [35] by knocking out the *EP153R* gene and the *EP402R*-*EP153R* and *UK* genes with the insertion of eGFP or dsRED reporter genes under the control of p72 promoters. Transfer plasmids were assembled from PCR fragments using the pUC19 vector (Thermo Fisher Scientific, Waltham, MA, USA) as the backbone for CRISPR/Cas9-mediated homologous recombination. For each transfer plasmid, two gRNA plasmids were produced by ligating one upstream and one downstream protospacer oligonucleotide into the BbsI-digested pX330-DNLS1_2-NeoR plasmid (Supplementary Table S1).

### 2.3. Production of the Recombinant ASFVs Stock

For stock preparation, 100 µL of the supernatant of isolated viruses was added to PAMs plated in 10 mL of culture medium on 75 cm^2^ flasks (SARSTEDT AG&Co. KG, Nümbrecht, Germany). The media of infected PAMs were harvested at three days post infection and titrated. Stock titers were determined by 10-fold serial dilution of the supernatants in the range of 10-1–10-7 in triplicate as described by Tamás et al. [36]. After immunofluorescence detection, positive cells in the lowest diluted wells were counted, averaged, multiplied by the dilution factor and expressed in fluorescent focus units (FFUs). Stock titers of all mutants remained in the range of 10^6^–10^8^/mL.

### 2.4. Animal Experiments

In vivo experiments were conducted under biosafety level 3 (BSL3) conditions at the animal facilities of Centro de Investigación en Sanidad Animal (CISA-INIA/CSIC) in accordance with the EC Directive 2010/63/UE and were approved by the Spanish Ethical and Animal Welfare Committee (Ref nº PROEX/101/8.21). All studies were conducted in ASFV-free and ASFV antibody-free 12-week-old European hybrid pigs, 20–25 kg weight, sourced from the same farm. We carried out three different in vivo experiments, in all of which all types of viruses were injected intramuscularly (IM). Virus/vaccine dilutions for in vivo vaccination/challenge were performed using macrophage medium.

Experiment 1: Twenty-one (21) domestic pigs were divided into four groups. Three groups of six pigs each were IM immunized with either 10^2^ TCID_50_ of Lv17/WB/Rie1-ΔEP153R (group 1.1), Lv17/WB/Rie1-ΔUK (group 1.2), or Lv17/WB/Rie1 parental strain (group 1.3, control). Three animals from group 1.4 served as unvaccinated controls. Thirty-five days after immunization (dpi), animals in group 1.1 and 1.4 were exposed to 10 HAD_50_ from Armenia/07 (Arm07) by IM route.

Experiment 2: Eighteen (18) domestic pigs were available for this study. Five animals per group received IM 10^2^ TCID_50_ of Lv17/WB/Rie1-ΔCD/ΔUK (group 2.1), Lv17/WB/Rie1-ΔCD (group 2.2), or the parental strain Lv17/WB/Rie1 (group 2.3). Three animals from group 4 served as unvaccinated controls. At 30 dpi, animals from all four groups were challenged with 10 HAD_50_ Armenia/07 (Arm07) via the IM route.

The design of the animal experiments is described in Table 1.

Experiment 3: The experiment aimed to explore the potential reversion of the Lv17/WB/Rie1-ΔCD MLV prototype to a more virulent form by five serial passages in domestic pigs. The study involved five different groups, designated as P1 through P5 comprising two 12-week-old pigs (identified as C1 through C10) each. Group P1 was IM inoculated with Lv17/WB/Rie1-ΔCD MLV at a dose of 10^4^ TCID_50_/mL. At 10 dpi, the two pigs in each group were euthanized, and samples were collected from 21 different organs, as outlined in Section 2.6. These samples were analyzed using the WOAH–Universal Probe Library (UPL) real-time PCR method [32] to detect the presence of the ASFV genome, following the procedures detailed in Section 2.6. The tissues exhibiting the highest viral genome loads were selected for further processing. These selected tissues were pooled in equal proportions and homogenized in phosphate-buffered saline (PBS) to create a 10% tissue suspension, as described in Section 2.6. The antibiotic-treated supernatants from this suspension were used as the inoculum for the subsequent group of pigs in the next passage. For P2, the tissue homogenate from P1 was back-titrated to 4.45 × 10^4^ TCID_50_/mL for inoculation. Similarly, the inoculum titers for the subsequent groups were adjusted as follows: P3 received 4.10 × 10^4^ TCID_50_/mL, P4 received 5.42 × 10^4^ TCID_50_/mL, and P5 received 4.92 × 10^4^ TCID_50_/mL. Tissue samples were collected at 10 dpi for each group from P1 to P5, and the tissues chosen for subsequent passages were always those with a cycle threshold (Ct) value of approximately 26.35 ± 0.4 (as shown in Table 2).

At the conclusion of the study, a comparative genome sequencing analysis was performed between the viral genomes of the pool of tissues obtained from P1, P4, and P5 in vivo passages (Section 2.7.2).

### 2.5. Sampling, Clinical, and Post-Mortem Examination

Clinical signs were recorded daily and expressed with a quantitative clinical score by summing the values of eight clinical signs as previously described by Gallardo et al. [34]. The clinical evaluation included parameters such as fever, anorexia, recumbency, skin hemorrhages or cyanosis, joint swelling, respiratory distress, ocular discharge, and digestive abnormalities. Each parameter was scored on a scale from 0 to 3, with 0 indicating no signs, 1 mild signs, 2 moderate signs, and 3 severe signs. The cumulative score for each pig, referred to as the clinical score (CS), was used to assess overall health and determine humane endpoints. Humane endpoints were established for pigs exhibiting severe clinical signs, including persistent fever, anorexia, recumbency, respiratory distress, or digestive symptoms for more than two consecutive days, or a CS exceeding 18.

EDTA blood and serum were collected once or twice per week from 0 dpi until the day of challenge (dpc) and twice per week after challenge until the last day of the experiment. During the experiments, unprotected pigs were euthanized at different time-points after reaching a specified endpoint, while all protected pigs were euthanized between 30 and 38 dpc. In experiment 3 of the reversion-to-virulence study, pigs from groups P1 to P5 were slaughtered at 10 dpi.

Gross lesions were evaluated in accordance with standardized protocols [36]. Twenty-one different types of tissues and organs were obtained from each necropsied animal; these were obtained from the liver; spleen; tonsil; heart; lung; kidney; submandibular, retropharyngeal, inguinal, popliteal, mesenteric, mediastinal, gastro-hepatic, splenic, and renal lymph nodes; bone marrow; diaphragmatic muscle; and intra-articular tissues of joints.

### 2.6. Laboratory Methods

#### 2.6.1. ASFV Genome Detection by Real-Time PCR

DNA was extracted from organ homogenates and blood samples using the High Pure PCR Template Preparation kit (Roche Diagnostics GmbH, Roche Applied Science, Mannheim, Germany). Briefly, 10% (*w*/*v*) clarified homogenized tissue suspensions were prepared in PBS using the TissueLyser II system. Tissue homogenates were centrifuged at 1800× *g* for 5 min. Supernatants were filtered with MINISART filters 0.45 μ and then treated with 0.1% of gentamicin sulfate 50 mg/mL (BioWhittaker, Walkersville, MD, USA) during 1 h at 4 ± 3 °C prior to use for virus detection. For the amplification of ASFV genomic DNA, WOAH-UPL real-time PCR [31] was carried out using undiluted extracted DNA for each sample. Samples with recorded Ct < 40.0 were considered positive, while samples with no recorded Ct value were considered negative.

#### 2.6.2. ASFV Detection by Virus Isolation

Virus isolation (VI) was conducted on PCR-positive blood and tissue samples using PBM cells seeded in 96-well plates. Plates were observed daily for seven days to detect HAD or cytopathic effect (CPE). If no HAD was observed, samples were subjected to three blind passages and then analyzed using UPL real-time PCR [31] to confirm the replication of non-HAD strains, including Lv17/WB/Rie1 or vaccine-derived strains. Viral titers were determined through end-point dilution assays performed on Cos7 cells, as detailed in Section 2.1.

#### 2.6.3. ASF Antibody Detection

The detection of ASFV-specific antibodies was carried out in serum samples using the IPT and the commercial ELISA kit (^®^INGENASA-INGEZIM PPA COMPAC K3, Gold Standard Diagnostic, Madrid, Spain). Tissue homogenates were also tested for antibody presence using the IPT. Antibody titers in all positive samples were quantified by end-point dilution via the IPT method [31].

### 2.7. Sequencing

#### 2.7.1. Sequencing of the ASFV Recombinant Viruses

Whole-genome sequencing of recombinant ASF viruses was performed according to Olasz et al. (2019) [37] on an Illumina platform (paired-end 150 bp). Sequence assembly was performed using Geneious Prime 2019.2.3. The Bowtie2 and BBMap mapping methods were used for analysis, with normal sensitivity. As a reference, LV17/WB/Rie1 was used. Virus sequences were deposited in the GenBank under the following accession numbers (acc. no.): Lv17/WB/Rie1-ΔEP153R, acc. no. PQ284532; Lv17/WB/Rie1-ΔUK acc. no. PQ284533; Lv17/WB/Rie1-ΔCD, acc. no. PQ284530; and Lv17/WB/Rie1-ΔCD/ΔUK, acc. no. PQ284531.

#### 2.7.2. Sequencing of the Samples Obtained from the Reversion-to-Virulence Study

The pool of tissues obtained from the P1, P4, and P5 in vivo passages were subjected to full-genome sequencing to study the genetic stability of the Lv17/WB/Rie1-ΔCD vaccine candidate. In detail, DNA was obtained and analyzed by PCR as described in Section 2.6.1. Libraries were prepared from a minimum starting amount of 100 ng of each input DNA with the NEBNext^®^ Ultra II™ FS DNA Library Prep Kit (New England BioLabs, Ipswich, MA, USA), each sample being labeled with a unique identifier. After a quality control step, DNA libraries were equivalently pooled in a single tube according to the ASFV quantity obtained by digital PCR using the primers and probe described by Fernández-Pinero et al., 2013, in the WOAH diagnostic manual [32]. An amount of 1000 ng of the pooled library was subjected to a targeted hybridization step using a custom-designed probe library which hybridizes selectively to the entire ASFV genome (ASFSeqCapv3.0 SeqCap EZ developer library, NimbleGen, Roche, Madison, WI, USA) and following the SeqCap EZ Library SR User’s Guide for Illumina. Afterwards, the enriched DNA library was purified, quality-checked on a TapeStation 4200 system (Agilent Technologies, Santa Clara, CA, USA), quantified with Quantus™ Fluorometer (Promega, Madison, WI, USA), and finally sequenced on an Illumina iSeq™ 100 system using iSeq 100 i1 Reagent v2 (300-cycle) to generate ~4 million paired-end reads (2 × 150 bp).

CLC Genomics Workbench (version 20.0.4) was used for data analysis. Initially, raw reads were checked for quality and trimmed. Reads were then mapped against the pig genome (GCA_000003025.6) to eliminate host contamination. Viral genomes were constructed by de novo assembly. Clean reads were mapped to the parental Lv17/WB/Rie1-ΔCD genome sequence (PQ284530) for variant calling (minimum read depth 20×; min. reads with variant 5×; min. variant freq 0.05). The identified variants were visually checked by IGV (Integrative Genomics Viewer, available at https://igv.org, accessed on 8 December 2024).

## 3. Results

### 3.1. Generation and Production of Lv17/WB/Rie1 Recombinant Viruses

The three single knockouts of the four deletion mutants (Lv17/WB/Rie1-ΔEP153R, Lv17/WB/Rie1-ΔCD, Lv17/WB/Rie1-ΔUK) were generated using CrispR/Cas9-facilitated ex vitro recombination by substituting large parts of the targeted genes with the p72 promoter-regulated eGFP or dsRED reporters in Lv17/WB/Rie1. The two CRISPR/Cas9 plasmids expressing the sgRNAs and Cas9 proteins and the transfer plasmids were co-transfected into Lv17/WB/Rie1-infected PAMs. The recombinant viruses were isolated from PAMs expressing the eGFP or the dsRED reporter proteins, respectively. After isolation, stocks were prepared and titered. Titers of the mutant viruses (Lv17/WB/Rie1-ΔCD: 2.4 × 10^7^ FFU/mL, Lv17/WB/Rie1-ΔEP153R: 3.8 × 10^7^ FFU/mL, Lv17/WB/Rie1-ΔUK: 1.1 × 10^7^ FFU/mL) somewhat exceeded that of the parental virus (7.2 × 10^6^ FFU/mL) grown under similar conditions. Lv17/WB/Rie1-ΔCD/ΔUK double knockout was created with a similar technique using Lv17/WB/Rie1-ΔUK as a parental strain and the same plasmids as those used for the ΔCD mutant. Its titer (6.74 × 10^6^ FFU/mL) was very similar to the wild-type virus.

The genome of the recombinant viruses was sequenced using the Illumina platform. Minimum 1.2 million reads were assembled and the average coverage (AC) of the genomes reached at least 750 in all cases (Lv17/WB/Rie1-ΔEP153R ~12 million reads, AC 8900; Lv17/WB/Rie1-ΔCD ~1.2 million reads, AC 750; Lv17/WB/Rie1-ΔUK 1.7million reads AC 1216; Lv17/WB/Rie1-ΔCD/ΔUK 38 million reads, AC ~34,000).The genome of all four viruses appears to be very stable in PAMs and shows very similar genetic variations compared to the parental wild-type virus genome, typically containing some mutations and ambiguous nucleotides in the inverted terminal region (positions 28., 34., 122., 130) and a few nucleotide indels in the poly C/G stretches (positions 15,666; 17,829; 19,992; 21,789) of the genomic 5′-end. In addition, the viruses have unique 1–2 nucleotide mutations, mostly in non-coding regions. The only major (six-nucleotide) insertion is found in a non-coding region of the Lv17/WB/Rie1-ΔCD/ΔUK virus (position 190,499) at the 3′ end of the genome (Table 3).

### 3.2. Deletion of the EP153R Gene Improves Safety While the Deletion of the UK Gene Increases the Virulence of the Parental Lv17/WB/Rie1 Strain

**Figure 1:**
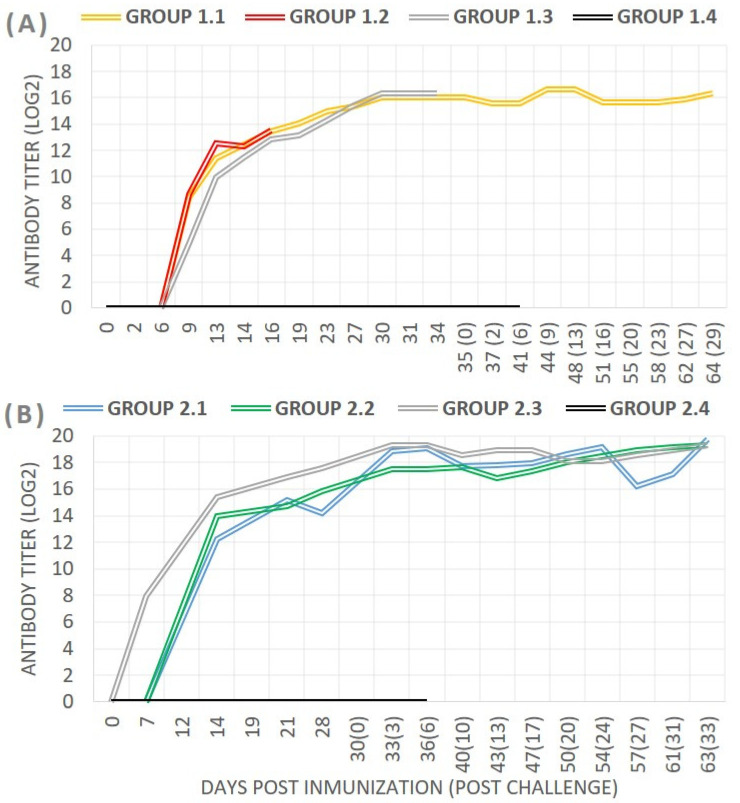
Detection of anti-ASFV antibodies in pigs after immunization and challenge with (**A**) Experiment 1: Lv17/WB/Rie1-∆EP153R (group 1.1), Lv17/WB/Rie1-∆UK (group 1.2), the parental strain (group 1.3), or unvaccinated (group 1.4) or (**B**) Experiment 2: Lv17/WB/Rie1-∆CD/∆UK (group 2.1), Lv17/WB/Rie1-∆CD (group 2.2), the parental strain (group 2.3), or the unvaccinated group (group 2.4). The antibody titers were assessed by IPT analysis and are expressed as the average daily Log10 per group.

*Antibody detection*: In Experiment 1, all pigs were efficiently immunized, as indicated by the antibody response, although slight differences were observed. In groups 1.1 and 1.2, vaccinated with the deletion mutants Lv17/WB/Rie1-∆EP153R and Lv17/WB/Rie1-∆UK, respectively, all pigs seroconverted from day 9, reaching similar antibody titers at 16 dpi of 13.42 ± 0.89 log2 (approximately 1.1 × 10^4^). In group 1.3 (Lv17/WB/Rie1), pigs developed antibodies from day 11 ± 1.6, reaching antibody titers of 10.99 ± 1.5 log2 (approximately 2.83 × 10^3^) at 16 dpi, one logarithm less than the other groups (Figure 1A). High antibody titers were present in the immunized, vaccinated, and challenged pigs until the end of the experiment.

*Clinical score and survival rate after vaccination:* Animals vaccinated with the Lv17/WB/Rie1-ΔUK deletion mutant (group 1.2) exhibited the highest mean clinical scores (7.16 ± 0.9) starting at 6 dpi, increasing to 12 points by day 13. All pigs in this group succumbed or were euthanized between 13 and 16 dpi due to severe adverse reactions, including sustained fever, erythema, hemorrhagic areas, and severe skin necrosis. The group immunized with the parental strain Lv17/WB/Rie1 (group 1.3) had a mean daily clinical score of 4.32 ± 1.31, with symptoms beginning on day 6–7 and progressively worsening, culminating in severe diarrhea and dehydration by day 34 associated with a secondary bacterial disease (*E. coli*), at which point all pigs were sacrificed. Group 1.1, vaccinated with Lv17/WB/Rie1-ΔEP153R, showed the lowest mean clinical scores (2.55 ± 2.61), with 66.6% (4/6) survival to the challenge day. Clinical signs appeared on day 7 and varied among animals, with two pigs (M1 and M5) developing severe symptoms with fever (mean 40.75 °C) from day 8–9 after vaccination, followed by the onset of lymphadenitis and joint inflammation, leading to euthanasia by day 19. The remaining four pigs, M2, M3, M4, and M6, did not present significant clinical signs and reached the challenge day with an average clinical score of 1.91 ± 0.7.

**Figure 2:**
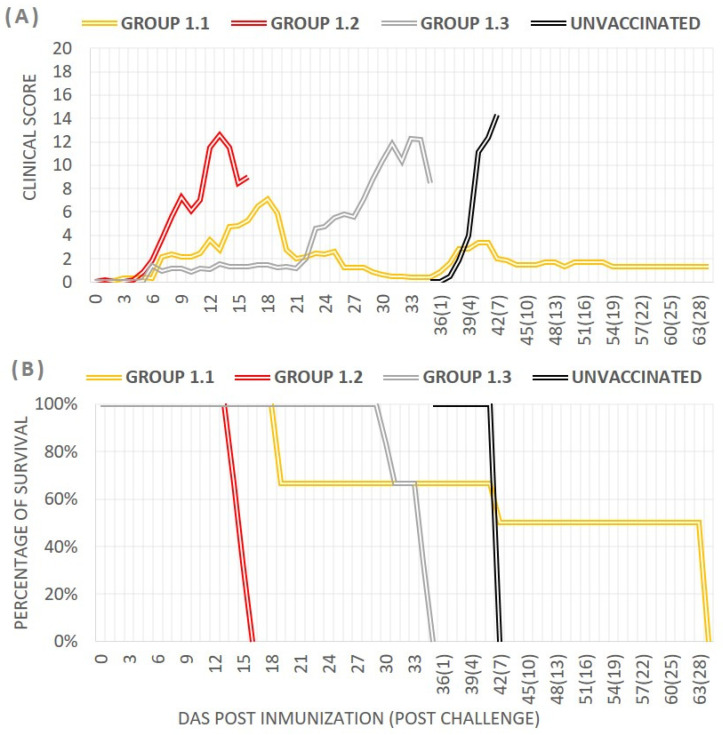
Average observed daily clinical scores (**A**) and percentage of survival (**B**) after immunization with Lv17/WB/Rie1-ΔEP153R (group 1.1), Lv17/WB/Rie1-ΔUK (group 1.2), or the parental Lv17/WB/Rie1 strain (group 1.3) and subsequent challenge at 35 dpi with Arm07. The black line represents the group of unvaccinated pigs.

Daily average clinical signs and survival rates after vaccination are shown in Figure 2.

**Figure 3:**
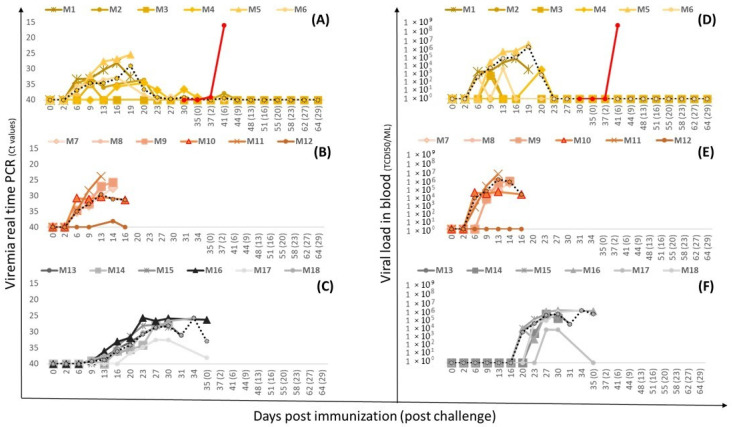
Viremia measured by real-time PCR and viral load in blood in pigs immunized with Lv17/WB/Rie1-∆EP153R (group 1.1, (**A**,**D**)), with Lv17/WB/Rie1-∆UK (group 1.2, (**B**,**E**)), or with the parental Lv17/WB/Rie1 strain (group 1.3, (**C**,**F**)). The dotted line represents the average daily viremia/virus titer. The red line in (**A**,**D**) represent the average daily viremia/virus titer in the group of unvaccinated pigs.

*Viremia and virus titer in blood:* Viremia varied among animals and correlated with clinical symptoms. In group 1.1 (Lv17/WB/Rie1-ΔEP153R), viremia began at 11 ± 4 dpi (mean daily Ct of 37.88 ± 1.63) and lasted an average of 19.8 days, with pig M3 having only a sporadic peak on day 9 (Figure 3A). Group 1.2 (Lv17/WB/Rie1-ΔUK) showed persistent viremia from day 6 with an average daily Ct of 31.88 ± 3.76, except pig M12, which had a sporadic peak on day 13 (Ct = 38.1) (Figure 3B). In the parental group (group 1.3), weak viremia started at 9 dpi in four pigs, reaching a mean Ct of 28.75 ± 2.37 at 27 dpi, coinciding with worsening clinical signs. Two pigs (M15 and M17) had late viremia between days 16 and 21, with M17 showing weak viremia and M15 displaying values similar to other pigs (Figure 3C).

Blood viral loads were assessed by the TCID_50_ assay in PBM (Figure 3D–F). In all pigs slaughtered before the challenge, the virus was isolated from PCR-positive blood samples from day 7.5 ± 1.3 until slaughter, with maximum viral titers of 9.18 × 10^5^ to 1.4 × 10^6^ TCID_50_/mL. In contrast, in the pigs that reached the challenge day within group 1, the virus was occasionally isolated from blood between days 6 and 20, with mean viral titers of 10^2^ TCID_50_/mL. In the parental group, infectious virus was detected from day 20 until sacrifice, with a maximum titer of 5.80 × 10^5^ TCID_50_/mL.

*Postmortem examination and virus load in tissues:* In group 1.1, two pigs died or were slaughtered prior to the challenge. Postmortem examinations revealed lesions consistent with chronic ASF, including severe joint inflammation, arthritis, and generalized lymphadenitis. Pig M5 exhibited the most severe lesions, such as fibrinous pericarditis and fibrinous-necrotizing pleuropneumonia affecting the apical, middle, and right caudal lung lobes. All samples tested positive for PCR and virus isolation (Supplementary Table S2).

All pigs in groups 1.2 and 1.3 were slaughtered before the challenge. In group 1.2, except for pig M12, animals vaccinated with the deleted UK gene displayed severe multifocal skin necrosis, pulmonary edema, hydrothorax, hydropericardium, and generalized lymphadenitis with enlarged, hemorrhagic lymph nodes, particularly the submandibular, retropharyngeal, inguinal, and mediastinal nodes. Virus isolation was successful in 90–100% of PCR-positive tissues, except for M12, where in 25% of tissues were infectious (Supplementary Table S2).

In group 1.3, lesions associated with chronic ASF included severe joint inflammation, arthritis, and generalized lymphadenitis without vascular disorders. Four out of six pigs showed digestive lesions, such as dilated, thin, and congested small intestines and yellow, watery diarrhea consistent with E. coli infection. Similar to group 1.2, ASFV was isolated from all PCR-positive samples, except for pig M17, which exhibited minimal ASF-compatible lesions and had the virus isolated from only 7 of 14 PCR-positive tissues or organs (Supplementary Table S2).

### 3.3. Pigs Vaccinated with Lv17/WB/Rie1-∆EP153R Were Protected Against Arm07 Challenge

To evaluate the efficacy of Lv17/WB/Rie1-∆EP153R, the four pigs in group 1.1 were IM inoculated at 35 dpi with Arm07. Three unvaccinated pigs (group 1.4) were included as controls. Pigs in the control group showed signs of ASF as early as 5 dpc, including high fever, anorexia, skin redness, prostration, and a staggered gait. They were sacrificed at 7 dpc with a mean CS of 14.33 ± 1.2 (Figure 2). On the day of euthanasia, the viral titer in the blood was 5.32 × 10^8^ HAD_50_/mL, with Ct values of 17.01 ± 0.8 (Figure 3A,D). All tissue samples tested positive for ASFV by PCR and viral isolation.

After the challenge, the pigs vaccinated with Lv17/WB/Rie1-∆EP153R did not show clinical signs and only pig M4 was euthanized at 6 dpc (41 dpi) due to clinical signs associated with lesions of the digestive tract with severe diarrhea, similar to that described in group 1.3 (Figure 2). This animal did not have viremia, and the lesions were consistent with chronic ASF infection and gastrointestinal bacterial disease. All tissues were confirmed positive by PCR, and the vaccine strain was isolated in 7 out of the 20 analyzed (35%) at 41 dpi (Supplementary Table S2). Among the other pigs, a low level of the ASFV genome was detected in the blood of one pig (M2) at 6 dpc (Figure 3A). The experiment ended with the euthanasia of all animals at 64 dpi (29 dpc) and the only notable lesions were found in pig M6 and were associated with mild lymphadenitis of the superficial lymph nodes. The ASFV genome was found in 6/21 tissues (28.6%) in the M3 pig, 7/21 (33.3%) in the M2 pig, and 11/21 (52.3%) in the M1pig. The virus was isolated from five tissues (23.8%) in pig M6 coinciding with the lesions (Supplementary Table S2). It is remarkable that the Arm07 virus was not isolated from any sample.

### 3.4. Deletion of the EP153R, CD2v, and UK Genes from Lv17/WB/Rie1, Either in Double (EP153R and CD2v) or Triple (EP153R, CD2v, and UK) Formats, Enhances Safety

*Antibody Response:* Similar to experiment 1, all pigs were efficiently immunized as demonstrated by the antibody response. All pigs immunized with the parental strain (group 2.3) seroconverted from day 7 onwards, while in the deletion mutant groups, antibodies were first detected on day 14. On the day of the challenge, antibody titers were in the range of 14.21 ± 1.87 log2 (≈5.3 × 10^5^) for group 2.1 (Lv17/WB/Rie1-ΔCD/ΔUK), 15.68 ± 0.55 log2 (≈1.71 × 10^5^) in group 2.2 (Lv17/WB/Rie1-ΔCD), and 17.66 ± 0.6 log2 (≈5 × 10^6^) in the parental group (Figure 1B). High antibody titers were present in the vaccinated and challenged pigs until the end of the experiment.

**Figure 4:**
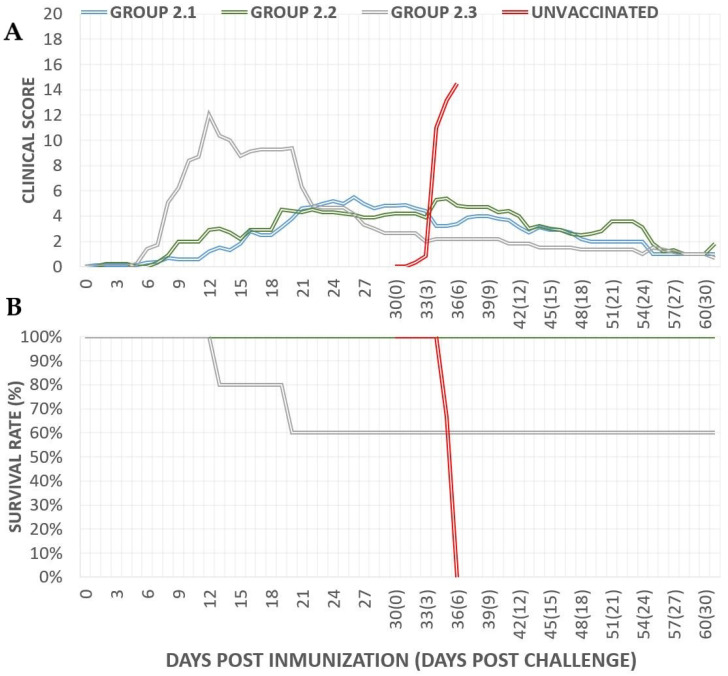
Average daily clinical scores (**A**) and percentage of survival (**B**) after immunization with Lv17/WB/Rie1-ΔCD/ΔUK (group 2.1), Lv17/WB/Rie1-ΔCD (group 2.2), or the parental Lv17/WB/Rie1 strain (group 2.3) and after challenge with Arm07. Unvaccinated animals are marked in red.

*Clinical signs and survival rate after vaccination*: Following vaccination, the parental group (group 2.3) exhibited an average daily clinical score of 5.2 ± 3.1, while the groups vaccinated with the mutant strains showed significantly lower scores: 2.46 ± 1.3 for group 2.1 (Lv17/WB/Rie1-ΔCD/ΔUK) and 2.37 ± 1.8 for group 2.2 (Lv17/WB/Rie1-ΔCD). In groups 2.1 and 2.2, vaccinated pigs developed mild clinical signs starting around day 7, gradually increasing to peaks of 5.5 points by day 26 in group 2.1 and 4.9 points by day 28 in group 2.2, until the challenge at 30 dpi (Figure 4A). In contrast, pigs immunized with the parental strain Lv17/WB/Rie1 (group 2.3) showed clinical signs at 5 dpi, which peaked at 13 points by day 12 before gradually declining (Figure 4A). Notably, two of the five pigs in this group died—one at 12 dpi and the other at 19 dpi—resulting in a 60% survival rate, whereas all pigs in the mutant groups survived until the challenge day (Figure 4B).

**Figure 5:**
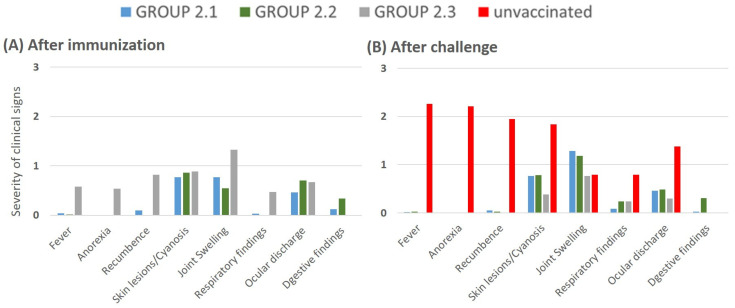
Severity of clinical signs observed in pigs after immunization (**A**) and challenge (**B**). The pigs were divided into groups: those vaccinated with Lv17/WB/Rie1-ΔCD/ΔUK (group 2.1), Lv17/WB/Rie1-ΔCD (group 2.2), or the parental Lv17/WB/Rie1 strain (group 2.3). The severity scale ranges from 1 (mild) to 2 (moderate), and from 2 to 3 (severe). Unvaccinated animals (group 2.4) are highlighted in red.

Pigs in groups 2.1 and 2.2 primarily exhibited mild clinical signs, with scores typically below 1. The only noticeable clinical sign was the development of tongue ulcers in four of the five pigs in group 2.1 during the second week, which lasted 5–7 days. In contrast, pigs immunized with the parental strain Lv17/WB/Rie1 (group 2.3) exhibited more severe clinical symptoms across various parameters, except digestive issues. In pigs C16 and C18, slaughtered at 12 and 19 dpi, respectively, rectal temperatures increased from 7.5 ± 1.8 dpi, peaking at 41.5 °C during the second week, coinciding with severe joint inflammation, generalized lymphadenitis, anorexia, and recumbence. The remaining pigs in this group experienced reduced temperatures and clinical signs after 14 dpi, with symptoms normalizing by the day of challenge. The severity of clinical signs observed in pigs after immunization is illustrated in Figure 5A.

**Figure 6:**
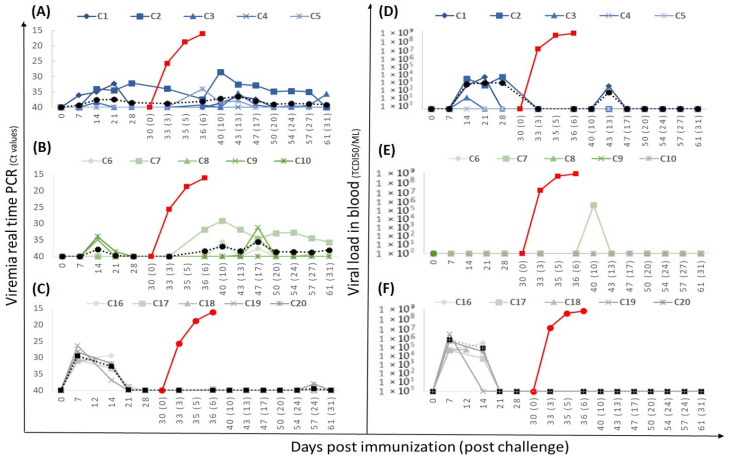
Viremia measured by real-time PCR and viral load in blood in pigs immunized with Lv17/WB/Rie1-ΔCD/ΔUK (group 2.1, (**A**,**D**)), Lv17/WB/Rie1-ΔCD (group 2.2, (**B**,**E**)), or the parental Lv17/WB/Rie1 strain (group 2.3, (**C**,**F**)) and after challenge with the Arm07-genotype II ASFV. The dotted line represents the average daily viremia/virus titer. The red line represent the average daily viremia/virus titer in the group of unvaccinated pigs.

*Viremia after vaccination:* In Group 2.1 (Lv17/WB/Rie1-ΔCD/ΔUK), weak and sporadic viremia was observed in three out of five animals (60%), beginning at 10.5 ± 4.95 dpi with a mean Ct value of 38.7 ± 1.4. Among these animals, C1 and C3 cleared the virus from their blood by 28 dpi, while C2 remained viremic. Virus isolation from blood samples was successful from day 14 to 21 (C1) and up to day 28 (C2), with an average titer of 9.74 × 10^2^ TCID_50_/mL (Figure 6A,D). In Group 2.2 (Lv17/WB/Rie1-ΔCD), two out of five animals (40%) exhibited transient viremia peak at 14 dpi (Figure 6B), but no infectious virus was detected prior to challenge (Figure 6E). In contrast, all five animals in Group 2.3 (vaccinated with the parental strain Lv17/WB/Rie1) showed peak viremia at 7 dpi, with a virus titer of 10^6^ TCID50/mL, which gradually cleared by 21 dpi and was absent by 28 dpi (Figure 6C,F).

### 3.5. Lv17/WB/Rie1-ΔCD/ΔUK and Lv17/WB/Rie1-ΔCD Provide Full Protection Against ASFV Genotype II

All pigs from groups 2.1 and 2.2 along with three pigs from the parental control group and the unvaccinated animals (group 2.4) were IM inoculated with the virulent Arm07 ASFV at dpi.

*Clinical signs and survival rate after challenge:* All vaccinated pigs, including those inoculated with either the deletion mutants or the parental strain, survived the challenge and showed 100% protection with minimal clinical signs. In contrast, the three unvaccinated pigs developed acute ASF by 3 dpc and were euthanized on day 6 (Figure 4A) after exhibiting severe symptoms, including high fever, anorexia, recumbency, skin lesions/cyanosis, and ocular discharge (Figure 5B). The vaccinated groups displayed a very low severity of symptoms overall, with the most notable sign being mild to moderate joint swelling, particularly in those immunized with the deletion mutants (Figure 5B). These clinical signs were similar to those observed during the vaccination period and gradually diminished over time. Other symptoms, such as fever, anorexia, and recumbence, were mostly absent.

*Viremia after challenge*: After challenge with Arm07 at 30 dpi, the data show a clear distinction between the vaccinated groups and the unvaccinated control pigs in terms of viremia and viral titer in the blood as is shown in Figure 6. The unvaccinated control pigs reached virus titers in blood of 6.26 × 10^8^ HAD_50_/mL on day 6 prior to slaughter, with Ct values of 16.15 ± 1.15. In contrast, no virus was detected post-challenge in pigs vaccinated with the parental strain (Group 2.3) by real-time PCR. In Groups 2.1 (Lv17/WB/Rie1-ΔCD/ΔUK) and 2.2 (Lv17/WB/Rie1-ΔCD), weak and sporadic viremia was detected in 3 out of the 5 animals. Only one pig per group (C2 and C7) displayed sustained viremia from days 6 to 10 post-challenge with a peak of viremia at 10 dpc reaching Ct values around 29. The vaccine virus was sporadically isolated from these animals on day 10 post challenge (Group 2.1) and day 13 post challenge (Group 2.2), with the highest titer of 2.12 × 10^5^ TCID_50_/mL found in Group 2.2 (C7). One month after the challenge, only one pig per group had weak viremia, with Ct values above 35.

*Postmortem examination and virus load in tissues:* In the parental Group 2.3, two pigs (C16 and C17) died prior to the challenge. Postmortem examinations revealed lesions consistent with chronic ASF, including severe joint inflammation, cutaneous ulcers, pericarditis, pneumonia, arthritis, and pleuritis. The remaining pigs from all three groups were slaughtered at the end of the study, between 31 and 38 days post-challenge (61 to 68 days after vaccination). No ASF-related lesions were found in any of these pigs.

Supplementary Table S3 shows the total number of positive PCR and virus isolation results for different tissue samples. In Group 2.1 (Lv17/WB/Rie1-ΔCD/ΔUK), PCR-positive samples ranged from 14.3% (C4) to 61.9% (C2), while virus isolation ranged from 4.7% (C2, C3, C4) to 14.3% (C1, C5). Notably, despite the absence of significant lesions, the virulent Arm07 virus was isolated from the renal lymph node of pig C2 and from the renal, gastro-hepatic, and submandibular lymph nodes of pig C5, with viral titers ranging from 6.25 × 10^3^ to 3.31 × 10^5^ HAD_50_/mL. In Group 2.2 (Lv17/WB/Rie1-ΔCD), the percentage of PCR-positive tissues ranged from 33.3% (C6, C10) to 70% (C8), with virus isolation rates ranging from 0% (C6, C7) to 14.8% (C8). Similarly to Group 2.1, the Arm07 virulent virus was isolated from the tonsils, renal, and submandibular lymph nodes of pig C7, with viral titers ranging from 2.77 × 10^3^ to 7.23 × 10^4^ HAD_50_/mL. In the parental strain group (Group 2.3), all tissues from pigs that died before the challenge (C16 and C18) tested positive for PCR, and virus was isolated from 17 out of 21 tissues in pig C16 (80.9%) and 100% of the tissues in pig C18. The highest viral titers were detected in the intra-articular tissues of joints, averaging 10 × 7 TCID_50_. In the remaining pigs (C17, C19, and C20), slaughtered at the end of the study, the percentage of PCR-positive tissues ranged from 28.6% (C17) to 47.6% (C19), with no virus isolated from these animals after the challenge. Non-immunized control pigs displayed characteristic ASF lesions after challenge. The ASFV genome was detected in all tissues, with an average Ct value of 21.30 ± 0.8. Virus isolation was successful from all samples, with mean titers ranging from 3.08 × 10^6^ HAD_50_/mL in intra-articular tissues of joints to 6.36 × 10^8^ HAD_50_/mL in target tissues such as the spleen.

### 3.6. Selection of Lv17/WB/Rie1-ΔCD MLV as the Optimal Vaccine Candidate: Comparative Effectiveness, Safety and Efficacy Parameters

Table 4 provides a comparative analysis of four Lv17/WB/Rie1 vaccine candidates with different gene deletions (ΔEP153R, ΔUK, ΔCD, ΔCD/ΔUK) against the parental strain, focusing on key parameters of effectiveness, safety, and efficacy.

Effectiveness was assessed through seroconversion rates and antibody titers measured on the final day before slaughter or challenge. All vaccine groups achieved 100% seroconversion, indicating a strong immune response. The highest average antibody titer was seen in the Lv17/WB/Rie1-ΔCD group (15.68 ± 0.55 log2), followed by the parental strain (15.16 ± 0.9 log2) and Lv17/WB/Rie1-ΔCD/ΔUK (14.21 ± 1.87 log2). Lower titers were observed in the Lv17/WB/Rie1-ΔUK (13.49 ± 2.12 log2) and Lv17/WB/Rie1-ΔEP153R (13.18 ± 1.26 log2) groups, with Lv17/WB/Rie1-ΔUK measurements taken at 14.5 ± 1.29 dpi due to early animal slaughter, possibly explaining the difference.

Safety was evaluated based on survival rates, clinical scores, and the presence of viremia following vaccination. The Lv17/WB/Rie1-ΔCD and Lv17/WB/Rie1-ΔCD/ΔUK groups demonstrated the highest safety, with 100% survival and low average clinical scores (2.37 ± 1.8 and 2.46 ± 1.3, respectively). The Lv17/WB/Rie1-ΔUK group had the poorest safety profile, with no survivors and the highest clinical scores (7.16 ± 0.9). Viremia was observed in all animals in the Lv17/WB/Rie1-ΔEP153R, Lv17/WB/Rie1-ΔUK, and parental strain groups. In contrast, viremia was sporadic in both the Lv17/WB/Rie1-ΔCD/ΔUK group (60%) and the Lv17/WB/Rie1-ΔCD group (40%).

Efficacy was measured by the protection rate after challenge, clinical scores, viremia, and the presence of the virulent strain in tissues. Both the Lv17/WB/Rie1-ΔCD and Lv17/WB/Rie1-ΔCD/ΔUK groups provided 100% protection against the ASFV challenge. The average post-challenge clinical scores were 3.26 ± 0.39 for the Lv17/WB/Rie1-ΔCD group and 2.70 ± 0.66 for the Lv17/WB/Rie1-ΔCD/ΔUK group, indicating mild to moderate symptoms. The parental strain also provided 100% protection, with slightly lower clinical scores (1.67 ± 0.40). Post-challenge viremia was minimal and sporadic in both mutant groups. The Lv17/WB/Rie1-ΔCD/ΔUK group showed the presence of the virulent strain in 40% of animals, with an average viral titer of 1.69 × 10^5^ HAD_50_/mL, while the Lv17/WB/Rie1-ΔCD group had it in 20% of animals, with a lower titer of 3.75 × 10^4^ HAD_50_/mL. No virulent strain was detected in the parental strain group.

### 3.7. Assessment of ASF Vaccine Candidate Lv17/WB/Rie1-ΔCD in a Reversion-to-Virulence Study

**Figure 7:**
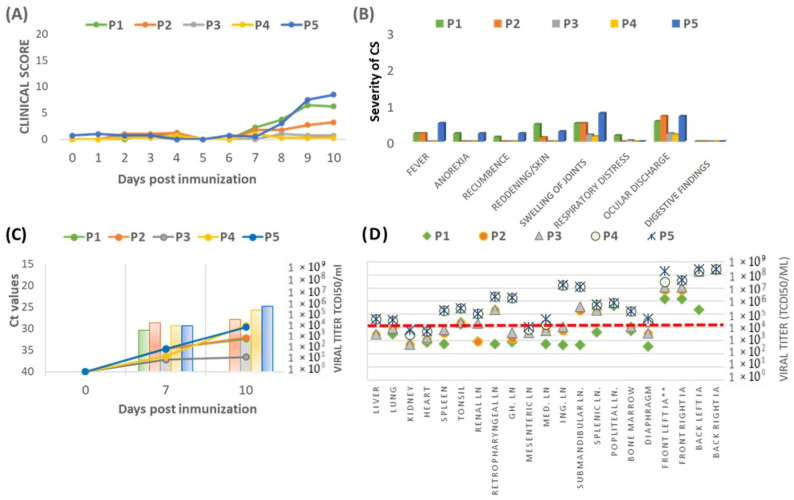
(**A**) Average daily clinical scores observed in pigs immunized with each passage. (**B**) Severity of clinical signs (CSs) observed in pigs after each passage. The severity scale ranges from 1 (mild) to 2 (moderate) and from 2 to 3 (severe). (**C**) Mean Ct values (left *y*-axis/lines) and viral titers (right *y*-axis/bars) measured 0, 7, and 10 days post-immunization (dpi) across five groups (P1–P5). (**D**) Mean viral titers in tissue samples measured at 10 dpi. LN = lymph nodes; IA = articular cartilage.

*In vivo study results:* An in vivo study was performed to evaluate the potential reversion to virulence of the Lv17/WB/Rie1-ΔCD vaccine candidate over five sequential passages in domestic pigs. In the group vaccinated with 10^4^ TCID_50_ of the prototype vaccine Lv17/WB/Rie1-ΔCD (P1), clinical signs appeared at 3 dpi and steadily increased, reaching a score of 6.25 by day 10. In the subsequent passages (P2 to P4), the severity of clinical signs progressively decreased, with the clinical score dropping to just 0.27 by 10 dpi in the P4 group. However, in the P5 group, a notable increase in clinical signs was observed starting at 8 dpi, peaking at a maximum score of 8.5 on day 10 (Figure 7A).

The severity of clinical signs across all groups generally remained mild, with values below 1 for most parameters (Figure 7B). However, in the fifth passage (P5 group), the severity of clinical signs increased, with symptoms such as fever, anorexia, recumbence, and joint swelling becoming notably more pronounced compared to the other groups. A notable increase in rectal temperature was observed in this group, with temperatures exceeded 41 °C by day 10 dpi. Skin reddening was most prominent in the P1 group, where only one pig developed a weak peak of fever between days 7 and 10, while in the P2 group, two pigs showed fever on days 9 and 10, though their temperatures did not exceed 40.5 °C. Ocular discharge severity was similar in groups P1, P2, and P5, with these groups showing higher values than P3 and P4. Animals in groups P3 and P4 exhibited only mild joint swelling and slight ocular discharge, without fever.

Viremia was detected in all groups by 7 dpi, with notable differences in viral titers and Ct values across the groups (Figure 7C). Despite the absence of significant clinical signs, the P4 group showed considerable viral replication by day 10, with viral titers reaching 1.39 × 10^5^ TCID_50_/mL (mean Ct = 29.32 ± 0.5), comparable to those in the P5 group, which had viral titers of 1.46 × 10^5^ TCID_50_/mL (mean Ct = 31.88 ± 3.0). The P2 group maintained viral titers below 10^5^ TCID_50_/mL, with a mean Ct value of 32.15 ± 3.1, while no virus was isolated from the P1 group by day 10, where only sporadic viral replication was observed at day 7. The P3 group had the lowest viral titers (9.26 × 10^2^ TCID_50_/mL) and mean Ct values (36.64 ± 1.7), indicating the least viral replication. Seroconversion occurred by 7 dpi in groups P1, P2, P3, and P5, but did not occur until 10 dpi in the P4 group.

On day 10, all pigs underwent a thorough pathological examination, with tissue samples analyzed by real-time PCR and virus isolation to determine viral load. In the P1 to P3 groups, only minor lesions were observed, including slightly enlarged lymph nodes and arthritis in the carpal and tarsal joints. The P4 group exhibited more significant lesions, such as petechiae in the urinary bladder lining, enlarged and hemorrhagic renal lymph nodes, slight hypertrophy of the interventricular septum, and highly congested and opaque atrioventricular and arterial valves, indicating cardiovascular involvement. The P5 group showed the most extensive pathological changes. This included moderate congestive-hemorrhagic foci in the splenic, gastro-hepatic, and renal lymph nodes, hemorrhagic foci in the kidney cortex, marked congestion in the kidney medulla, extensive erosions in the stomach’s fundic mucosa, and thickened mucosa in both the small and large intestines. The lungs had consolidation foci, particularly in the dorsal portion of the right lung, suggesting localized pneumonia. The pericardium contained abundant fibrin deposits, and the heart showed congestive coronary areas in the myocardium and congestive endocarditis in the atrioventricular and arterial valves. Additionally, the mediastinal lymph nodes showed congestive foci.

The highest viral titers were observed in the intra-articular cartilage of the front and back joints on both sides in all groups, exceeding 10^5^ TCID_50_/mL (Figure 7D). In the P5 group, these titers reached up to 10^9^ TCID_50_/mL, while the P4 group showed slightly lower titers, ranging from 10^7^ to 10^8^ TCID_50_/mL. Other tissues, including the spleen, tonsils, and various lymph nodes, also had substantial viral titers in the P4 and P5 groups, often above 10^5^ TCID_50_/mL. In contrast, the P2 and P3 groups had viral titers below 10^5^ TCID_50_/mL, except for certain lymph nodes, which ranged from 10^5^ to 10^6^ TCID_50_/mL. The P1 group consistently showed the lowest viral titers, around 10^4^ TCID_50_/mL, across all tissues.

*Genetic stability results*. The pool of tissues obtained from groups P1, P4, and P5 were selected to study the in vivo genetic stability of the Lv17/WB/Rie1-ΔCD vaccine candidate. DNA reads from the whole-genome sequencing of the three passage samples were mapped to the parental Lv17/WB/Rie1-ΔCD genome, obtaining a mean coverage from 750× to 862×. Because of the high read depth obtained along the genome, a number of variants (SNPs and indels) were revealed for each passage. Specifically, 214, 115, and 247 variations, complying with the parameters considered for the variant calling (freq. > 0.05), were reported for each P1, P4, and P5, respectively. These variant numbers were reduced to 26, 21, and 27 for a frequency over 10%. Variant calling analysis revealed a very short number of main variations (freq. > 0.5) along the genome (Supplementary Table S4). Specifically, only one deletion of 1–2 nucleotides was identified in the three P1, P4, and P5, located within the MGF-110-*14L* coding region, potentially producing a frameshift. This particular region corresponds to a large C homopolymer which is well known to be challenging to resolve by NGS, so this deletion may not be true. Interestingly, this homopolymer contains 9C in the reference Lv17/WB/Rie1 strain (Table 5). Even more, the variant frequency is rather similar in P1 and P4, where it is 72.46% and 80.94% (41.93% 1Cdel + 39.01% 2Cdel), respectively, and is close to 50% in P5 (56.46%); all these observations may support the hypothesis of a false-called deletion (Supplementary Table S4).

In addition, three main single-nucleotide mutations were identified after only five passages (P5). In detail, transition G→A occurred at the 8399 nt position, located in the intergenic region between MGF-110-*2L* and MGF-110-*3L*. Two transitions C→T were identified within MGF-300-1L coding gene. From these, the mutation that happened at 21,364 nt position did not produce any protein modification, while the variation at 21,450 nt position resulted in the amino acid change A88T in the protein sequence. Interestingly, among the previous in vivo passages, only P4 displayed the two transitions in MGF-300-*1L* as minor variants, with a frequency below 10% (Table 5).

## 4. Discussion

One promising candidate in ASFV vaccine development is the naturally attenuated strain Lv17/WB/Rie1, which has shown effective protection in wild boar through oral immunization against homologous virulent challenges [20]. However, its application as a vaccine strain for domestic pigs is limited due to cause mild symptoms, such as joint swelling and ear cyanosis [21]. Additionally, the strain lacks DIVA capabilities, making further modifications necessary before it can be widely used as a safe and effective vaccine. The challenge lies in identifying which viral genes should be deleted to enhance vaccine safety, as this varies depending on the ASFV strain, making gene selection critical for both safety and effectiveness [38,39]. For instance, deleting the *EP402R*, *EP153R*, and *BL119* (9GL) genes from the Lv17/WB/Rie1 isolate did not affect the virus’s attenuated phenotype but resulted in a loss of protection compared to the parental strain [40]. Conversely, deletion of the MGF 110-*11L* gene increased the virus’s virulence [35]. Therefore, gene deletions must be carefully chosen to maintain the delicate balance between safety and protection [39,40].

This study aims to further reduce the residual virulence of Lv17/WB/Rie1 and enhance the safety of the vaccine candidate by deleting genes. To optimize these vaccine candidates, a positive marker was introduced in the single-, double-, or multiple-gene-deleted prototypes to create a serological DIVA-compatible vaccine. All experiments were performed using the intramuscular route for its ability to ensure precise dosing, efficient antigen delivery, and reduced variability, making it ideal for proof-of-concept studies aiming to evaluate vaccine efficacy under controlled conditions. In the initial experiments, we evaluated the safety and efficacy of Lv17/WB/Rie1 by individually deleting the UK (*DP96R*) gene [29,30,39] or the *EP153R* gene [25,26,27]. All groups immunized with the recombinant viruses produced high specific antibody levels from day 7–9 post-infection, similar to the parental strain. However, despite high antibody titers in the Lv17/WB/Rie1-ΔUK group, the UK gene deletion unexpectedly increased virulence, with pigs showing severe symptoms, high clinical scores, and early euthanasia by day 16 post-immunization. This finding underscores the strain-dependent nature of ASFV virulence, as *UK* gene deletion has shown varying effects across different ASFV strains [30]. For example, deleting the *UK* gene from ASFV strain E70 significantly reduced virulence, whereas its removal from the ASFV-G (Georgia strain) did not [41]. In this study, the heightened immune response was insufficient to mitigate the increased virulence, indicating that the single *UK* gene deletion is not suitable for vaccine development without further modifications.

Although weak viremia was detected in the parental strain group from day 9, viral titers increased as clinical signs worsened over time, leading to the slaughter of all animals prior to the challenge. This aligns with previous studies suggesting that while the Lv17/WB/Rie1 strain is attenuated, it retains some residual virulence, which may manifest under certain conditions, such as secondary infections (e.g., *E. coli*), as seen in this group [20].

Pigs vaccinated with Lv17/WB/Rie1-ΔEP153R exhibited the lowest clinical scores and the highest survival rate (66.6%) after vaccination, although two animals developed severe clinical signs and required euthanasia on day 19 post-vaccination. It is noteworthy that the Lv17/WB/Rie1-ΔEP153R mutant provided complete protection against Arm07 challenge in all remaining pigs. Three out of four pigs survived the challenge without displaying either clinical signs or viremia and ASFV was not isolated from any samples after the challenge. This confirms that the *EP153R* deletion does not compromise the vaccine’s protective efficacy and maintains the attenuated profile of the Lv17/WB/Rie1 strain [20], in contrast to that observed with the non-HAD and attenuated genotype I isolate ASFV/NH/P68 [34]. Additionally, *EP153R* plays a critical role in hemadsorption inhibition (HAI) and serves as a marker for developing companion DIVA tests, making *EP153R* a promising candidate for further DIVA vaccine development [42,43,44].

Previous studies suggest that targeting multiple genes in combination is more effective in reducing virulence while maintaining protective efficacy [12,39,40]. The *EP402R* gene, which encodes the CD2v protein, is a key candidate for deletion due to its role in viral dissemination by allowing infected cells or viral particles to bind to red blood cells (RBCs) in pigs [39]. CD2v’s importance in protection is supported by the findings that (i) immunization with recombinant CD2v provides partial protection [41], (ii) sera that inhibit RBC binding correlate with cross-protection between serogroups [26,27,43], and (iii) CD2v contains both T- and B-cell epitopes [27]. To prevent reversion to a more virulent form, we fully deleted the *EP402R* gene from the Lv17/WB/Rie1-ΔEP153R virus, as this gene is not essential for virus replication. Previous research also shows that deleting *EP402R* and *EP153R* together further reduces clinical symptoms, virulence, and viral persistence in the blood [44,45].

To address this, we introduced a third gene deletion, targeting the UK gene. Previous studies demonstrated that deleting the UK gene from a genotype II EP402R/CD2v-deleted Chinese isolate (ASFV-SY18) resulted in greater attenuation and conferred 100% protection in pigs [46]. Although, in this study, the single deletion of the *UK* gene unexpectedly increased virulence, its role as a modulator of type I IFN and pro-inflammatory responses by blocking IRF3 nuclear translocation [47] makes it an important target for combined gene deletions. Thus, by adding the UK gene deletion to the *EP402R* and *EP153R* deletions, we aim to further enhance attenuation while ensuring robust protective efficacy, leading to the development of a safer and more effective vaccine candidate. This formed the basis for our second experiment, where we evaluated the safety and efficacy of the double *E153R*/*EP402R* (named ∆CD) and the triple (∆CD-UK) mutants in comparison to the parental Lv17/WB/Rie1 strain.

Similar to the results observed in experiment 1, all pigs vaccinated with the deletion mutants (Lv17/WB/Rie1-ΔCD and Lv17/WB/Rie1-ΔCD/ΔUK) developed a strong and sustained humoral response throughout the study similar to that observed in the parental strain. During the vaccination phase, pigs immunized with both the double deletion (Lv17/WB/Rie1-ΔCD) and the triple deletion (Lv17/WB/Rie1-ΔCD/ΔUK) mutants experienced weaker and more sporadic viremia compared to those vaccinated with the parental strain. This lower viremia corresponded with significantly reduced clinical signs, with daily clinical scores below 2.5 and the absence of severe symptoms such as high fever. In contrast, pigs vaccinated with the parental strain exhibited more severe clinical outcomes, including joint inflammation, skin ulcers, and lymphadenitis, leading to two fatalities and a 60% survival rate by the end of the vaccination phase, consistent with previous studies [20].

Following the challenge with Arm07, the unvaccinated control pigs developed acute ASF, displaying severe clinical signs and extensive viral dissemination. Viral titers in these animals were exceptionally high, reaching up to 6.36 × 10^8^ HAD_50_/mL in key tissues such as the spleen, and the ASFV genome was detected in all tissues. In contrast, the deletion mutant groups (Lv17/WB/Rie1-ΔCD and Lv17/WB/Rie1-ΔCD/ΔUK) showed a marked reduction in viral replication and disease severity. All pigs vaccinated with the deletion mutants survived the challenge, displaying only mild clinical signs, such as joint swelling, which diminished over time. Viremia was weak and sporadic, and virus shedding via the bloodstream was significantly reduced, with only isolated cases showing low viral titers of vaccine strain. PCR analysis revealed lower viral persistence, with PCR-positive tissues ranging from 14.3% to 61.9% in Group 2.1 (Lv17/WB/Rie1-ΔCD/ΔUK) and from 33.3% to 70% in Group 2.2 (Lv17/WB/Rie1-ΔCD). Although the virulent Arm07 virus was isolated from lymph nodes in these groups, the viral titers were dramatically lower than in unvaccinated controls. While these findings demonstrate that the deletion mutants drastically reduce acute infection and viral shedding, the presence of low viral titers in some tissues suggests the potential for virus persistence. Similar observations have been made with other ASFV vaccine candidates, such as “ASFV-G-∆MGF”, where traces of challenge virus replication were detected in a single animal using differentiated real-time PCR, despite full clinical protection [15]. Moreover, this low-level persistence in certain tissues raises the possibility of viral shedding under specific conditions, particularly in field environments where external factors, such as animal interactions, could influence transmission dynamics and recombination events in the field [17,48].

For the comparative in vivo studies, the Lv17/WB/Rie1-ΔCD vaccine candidate was selected as the optimal choice based on its superior balance of effectiveness, safety, and efficacy compared to the other mutants. The Lv17/WB/Rie1-ΔCD group demonstrated the highest average antibody titers, indicating a robust immune response, and achieved 100% seroconversion. In terms of safety, the Lv17/WB/Rie1-ΔCD group exhibited lowest clinical scores and sporadic viremia without vaccine shedding, which suggests a reduced risk of adverse effects. Moreover, the Lv17/WB/Rie1-ΔCD vaccine provided complete protection against the Arm07 challenge with minimal post-challenge clinical symptoms and lower viral titers in tissues, further supporting its effectiveness. These features position the Lv17/WB/Rie1-ΔCD candidate as a leading option for a live attenuated vaccine. For this reason, it was selected for a further reversion-to-virulence study in domestic pigs.

However, despite the promising results, the reversion-to-virulence study revealed some concerns. In the course of five passages in pigs, Lv17/WB/Rie1-ΔCD showed mild clinical signs in most groups (passages P1–P4), but a noticeable increase in clinical severity was observed in the last group, P5, where animals exhibited pronounced symptoms such as fever, joint swelling, and higher viral titers in various tissues, especially the joints. The P5 group’s heightened clinical signs and viral replication suggest that while the Lv17/WB/Rie1-ΔCD candidate is generally safe, there remains a potential risk of reversion to virulence after multiple in vivo passages. The reversion was also supported by the genetic analysis, which identified a small number of genetic variations. The sequencing data revealed a potential frameshift in the MGF-110-*14L* gene due to a deletion of 1–2 nucleotides. This deletion was identified in passages P1, P4, and P5, which may result in a frameshift mutation that could impact the function of the encoded protein. However, this region contains a large homopolymer (a sequence of nine consecutive cytosines), which is known to be prone to sequencing errors by NGS technologies. Additionally, the consistent frequency of this deletion across the passages suggests this could be a false-called deletion rather than a true genetic variation. Remarkably, two main mutations were observed in the MGF300-1L gene after five passages in vivo. Specifically, two transitions (C→T) were identified within this gene, one of which (at nucleotide position 21,450) led to an amino acid change (A88T), potentially altering the protein’s function. The MGF300 family genes (MGF300-*1L*, MGF300-*2R*, and MGF300-*4L*) have been shown to significantly affect ASFV replication in PAMs [49], which are key target cells for ASFV infection. The mutations in MGF300-1L may influence the replication dynamics of the virus, potentially contributing to the reversion to virulence observed in the P5 group. The MGF300 family plays a critical role in modulating the virus’s ability to replicate efficiently within the host’s immune cells. Previous studies have shown that deletions or alterations in these genes can attenuate the virus, reducing its replication in PAMs, and in some cases, lead to a less virulent phenotype [49,50]. However, mutations in these genes, as observed in the P5 passage, could potentially enhance replication or restore virulence, which aligns with the increased clinical severity and viral load seen in the P5 group. The specific mutation in MGF300-*1L* observed here may have contributed to the enhanced replication and virulence seen in later passages, emphasizing the importance of this gene family in controlling ASFV pathogenicity. While the potential frameshift in MGF-110-*14L* is likely a sequencing artifact, the mutations in MGF300-*1L* could be biologically relevant and may have influenced the observed reversion to virulence by enhancing viral replication in key immune cells like PAMs. Further functional studies are needed to fully understand the impact of these genetic changes on the virus’s behavior.

In summary, the Lv17/WB/Rie1-ΔCD candidate demonstrates strong protection and a favorable safety profile, but the reversion study indicates the need for further optimization to ensure long-term stability. Historical challenges with ASF vaccines, such as those in Spain, Portugal, and more recently in China, have shown that rushing vaccine deployment can lead to prolonged outbreaks, with animals displaying delayed symptoms and extended virus shedding [51]. This underscores the importance of ensuring that any ASF vaccine is stable and avoids reversion to virulence or recombination with field strains, which could exacerbate the spread of the disease. The potential risk of recombination with circulating wild-type strains [18,48], though low, reinforces the importance of deploying live vaccines only under controlled conditions with minimal ASFV circulation. Strict biosecurity, enhanced surveillance, and careful monitoring are essential to mitigate these risks and prevent the emergence of new viral variants with unpredictable characteristics.

An essential component of ASF vaccination is the DIVA test, which enables authorities to distinguish between vaccinated and naturally infected animals. This is of great importance for effective vaccination programs and to quickly identify potential issues. However, current ASF vaccines, introduced in Vietnam between 2022 and 2023, face limitations in this regard, despite recent attempts to develop a serological DIVA marker [52]. While they are technically DIVA-compatible due to the deletion of a specific gene, the low-intensity and sporadic viremia they induce limits differentiation to highly specialized laboratories, creating uncertainty about their compliance with WOAH standards. The Lv17/WB/Rie1-ΔCD candidate offers a significant advantage in this area by utilizing the deletion of the *EP153R* gene as a negative marker, ensuring easy differentiation between vaccinated and infected animals. A reliable DIVA test, developed in parallel with this study (Patent EP22462010.4), uses the deleted EP153R as a marker, combined with a reporter marker gene (eGFP), making the vaccine strain easily identifiable through molecular and serological methods [53]. This design ensures a clear distinction between the vaccine strain and natural ASFV strains.

Despite these promising features, the reversion-to-virulence study highlights the importance of continued improvements. The observed increase in clinical signs and viral replication in the P5 group suggests a potential risk of reversion after multiple passages. Therefore, further refinement and long-term evaluation are necessary to ensure the genetic stability and safety of the vaccine strain. With these enhancements, the Lv17/WB/Rie1-ΔCD candidate could become a highly effective and reliable vaccine strain for ASF control, meeting international standards for disease prevention.

## 5. Conclusions

The Lv17/WB/Rie1-ΔCD vaccine candidate, administered as a single-dose intramuscular immunization, shows strong potential, providing robust protection and a favorable safety profile compared to the parental Lv17/WB/Rie1 strain. The deletion of *EP153R* and *EP402R* enhances vaccine safety while offering DIVA compatibility, making it a promising tool for ASF control. However, the reversion-to-virulence study highlights concerns about genetic stability after multiple in vivo passages, at least under the experimental conditions used in this pilot study, indicating a need for further optimization. Continued refinement and long-term evaluation are essential to ensure safety and prevent reversion, but with these improvements, the Lv17/WB/Rie1-ΔCD candidate could serve as a safe, effective, and internationally compliant ASF vaccine.

## 6. Patents

International application No. PCT/EP2023/082518 for “ATTENUATED AFRICAN SWINE FEVER VIRUS AND USE THEREOF IN VACCINE COMPOSITIONS” in the name of INTERVET INTERNATIONAL B.V., CONSEJO SUPERIOR DE INVESTIGACIONES CIENTÍFICAS (CSIC), ÁLLATORVOSTUDOMÁNYI KUTATÓINTÉZET, UNIVERSIDAD COMPLUTENSE DE MADRID and GOLD STANDARD DIAGNOSTICS MADRID, S.A.

## Figures and Tables

**Table 1:** Design of the animal experiments.

Experimental Groups	ID VACCINE	ID ANIMAL
GROUP 1.1	Lv17/WB/Rie1-ΔEP153R	M1, M2, M3, M4, M5, M6
GROUP 1.2	Lv17/WB/Rie1-ΔUK	M7, M8, M9, M10, M11, M12
GROUP 1.3	Lv17/WB/Rie1	M13, M14, M15, M16, M17, M18
GROUP 1.4	Unvaccinated	M19, M20, M21
GROUP 2.1	Lv17/WB/Rie1-ΔCD/ΔUK	C1, C2, C3, C4, C5
GROUP 2.2	Lv17/WB/Rie1-ΔCD	C6, C7, C8, C9, C10
GROUP 2.3	Lv17/WB/Rie1	C16, C17, C18, C19, C20
GROUP 2.4	Unvaccinated	C21, C22, C23

**Table 2:** Overview of serial passages in the reversion-to-virulence study of Lv17/WB/Rie1-ΔCD in domestic pigs describing the tissue pooling, Ct values, and doses of the inocula.

Group	ID Pigs	Vaccination		
		Inocula	Ct Average	Dose (TCID_50_) per Animal/IM Route
P1	C1, C2	Lv17/WB/Rie1-ΔCD	26.88	10^4^
P2	C3, C4	Pool of 4 tissues from P1 (front left IA * C1, front right IA C2, back left IA C2 and popliteal LN **)	26.37 ± 0.9	4.45 × 10^4^
P3	C5, C6	Pool of 4 tissues from P2 (front right IA C3, submandibular LN C3 and popliteal LNs C3 and C4)	27.06 ± 3.8	4.10 × 10^4^
P4	C7, C8	Pool of 5 tissues from P3 (popliteal LNs C5, gastro hepatic and submandibular LNs C6, front left IA * C6 and front right IA C6)	25.93 ± 2.9	5.42 × 10^4^
P5	C9, C10	Pool of 4 tissues from P4 (tonsil C7, retropharyngeal LNs C7 and C8 and splenic LN, C8)	26.15 ± 1	4.92 × 10^4^

* IC; articular cartilage; ** LN: lymph node.

**Table 3:** Sequence differences in the four Lv17/WB/Rie1 mutants. Numbering is according to Lv17/WB/Rie1 positions.

Recombinant Virus	Nucleotide Position in Lv17/WB/Rie1	Mutation Type	Change	Type of Change
Lv17/WB/Rie1-ΔEP153R	43	point mutation	T->C	NCR
	1022	deletion	delG	NCR
	156,66	insertion	insC (9->10C)	frameshift MGF 110-14L
	73,875–74,209	planned genetic modification	EP153R->pp72-eGFP	Transgene
	181,933	point mutation	A->G	synonym, i7L
Lv17/WB/Rie1-ΔCD	43; 122; 130	ambiguity	Y, S, W	NCR
	1022	deletion	delG	NCR
	14,236	deletion	deletion C (50%) (10->9C)	potential frameshift MGF 110-11L
	15,666	insertion	ins2C (9C->11C)	frameshift MGF 110-14L
	73,812–75,384	planned genetic modification	EP153R and EP402R->pp72-eGFP	Transgene
	189,481	insertion	insG (8C->9C)	NCR
Lv17/WB/Rie1-ΔUK	43; 122; 130	ambiguity	Y, S, W	NCR
	14,236	deletion, ambiguity	deletion (50%) (10->9C)	potential frameshift MGF 110-11L
	15,666	insertion	insC (9C->10C)	frameshift MGF 110-14L
	17,829	insertion	insG (10C->11C)	NCR
	21,789	insertion	ins2G (8C->10C)	NCR
	185,514–185,623	planned genetic modification	DP96R->pp72-dsRED	Transgene
Lv17/WB/Rie1-ΔCD/ΔUK	28; 34; 122; 130	ambiguity	W, S, S, Y	NCR
	15,666	insertion	ins3C (9->12C)	frameshift MGF 110-14L
	17,829	insertion	ins2G (10C->12C)	NCR
	19,992	insertion	ins2G (9C->11C)	NCR
	73,812–75,384	planned genetic modification	EP153R and EP402R->pp72-eGFP	Transgene
	185,295–185,623	planned genetic modification	DP96R->pp72-dsRED	Transgene
	190,499	insertion	CAGTAG	NCR

**Table 4:** Comparative evaluation of the safety, antibody response, and protective efficacy of Lv17/WB/Rie1 vaccine candidates with targeted gene deletions.

		Experiment 1			Experiment 2		
PARAMETER versus MLV		Lv17/WB/Rie1-ΔEP153R (Group 1.1)	Lv17/WB/Rie1-ΔUK (Group 1.2)	Parental Lv17/WB/Rie1 (Group 1.3)	Lv17/WB/Rie1-ΔCD/ΔUK (Group 2.1)	Lv17/WB/Rie1-ΔCD (Group 2.2)	Parental Lv17/WB/Rie1 (Group 2.3)
Effectiveness	SEROCONVERSION	100% (6/6)	100% (6/6)	100% (6/6)	100% (5/5)	100% (5/5)	100% (5/5)
	First antibody positive finding _[±SD]_	9 dpi	9 dpi	11 ± 1.6 dpi	14 dpi	14 dpi	7 dpi
	AVERAGE Ab titer *.	13.18 ± 1.26 log2	13.49 ± 2.12 log2	15.16 ± 0.9 log2	14.21 ± 1.87 log2	15.68 ± 0.55 log2	17.66 ± 0.6 log2
Safety	SURVIVAL AFTER VACCINATION	66.6% (4/6)	0% (0/6)	0% (0/6)	100% (5/5)	100% (5/5)	60% (3/5)
	AVERAGE CLINICAL SCORE	2.55 ± 2.61	7.16 ± 0.9	4.32 ± 1.31	2.46 ± 1.3	2.37 ± 1.8	5.2 ± 3.1
	Mean time to death _[±SD]_	21 ± 2.7 dpi	14.5 ± 1.29 dpi	34 dpi	-	-	15 ± 2.44 dpi
	VIREMIA AFTER VACCINATION	100% (6/6)	100% (6/6)	100% (6/6)	60% (3/5)	40% (2/5)	100% (5/5)
	First PCR-positive finding _[±SD]_	11 ± 4 dpi	6 dpi	15 ± 3.89 dpi	10.5 ± 4.95 dpi	14 dpi (sporadic)	7 dpi
	AVERAGE daily Ct	37.88 ± 1.63	31.88 ± 3.76	32.56 ± 3.89	38.7 ± 1.4	39.5 ± 1.4	36.4 ± 0.6
	VIRAL REPLICATION (BLOOD)	83.3% (5/6)	83.3% (5/6)	100% (6/6)	60% (3/5)	0% (0/5)	100% (5/5)
	AVERAGE daily titer (TCDI_50_/mL)	2.72 × 10^2^	2.92 × 10^5^	1.28 × 10^5^	5.85 × 10^2^	Neg	9.99 × 10^4^
	VIRAL REPLICATION WINDOW **	6–21 dpi	6–16 dpi	20–35 dpi	14–28 dpi	Neg	7–14 dpi
Efficacy	PROTECTION RATE	75% (3/4)	-	-	100% (5/5)	100% (5/5)	100% (3/3)
	AVERAGE CS	2.25 ± 1.62	-	-	2.70 ± 0.66	3.26 ± 0.39	1.67 ± 0.40
	Mean time to death _[±SD]_	6 dpc	-	-	-	-	-
	VIREMIA AFTER CHALLENGE	25% (1/4)	-	-	80% (4/5)	60% (3/5)	0% (0/3)
	ONSET OF VIREMIA by PCR	Sporadic (6 dpc)	-	-	17.5 ± 10.6	13.5 ± 10.7	Neg
	AVERAGE daily Ct	37.9	-	-	38.4 ± 1	39.5 ± 1.4	Neg
	VIRAL REPLICACTION (BLOOD)	0% (0/4)	-	-	20% (1/5)	20% (1/5)	0% (0/3)
	AVERAGE daily titer (TCDI_50_/mL)	Neg	-	-	4.45 × 10^2^	2.12 × 10^5^	Neg
	VIRAL REPLICATION WINDOW **	Neg	-	-	13 dpc	10 dpc	Neg
	VIRAL REPLICATION (TISSUES)	0% (0/2)	-	-	100% (5/5)	75% (3/5)	0% (0/3)
	PRESENCE OF VIRULENT STRAIN	-	-	-	40% (2/5)	20% (1/5)	No
	Average viral titer (HAD_50_/mL)	-	-	-	1.69 × 10^5^	3.75 × 10^4^	-

CS = clinical score; dpi = days post immunization; dpc = days post challenge. * Measured on the final day before slaughter or the challenge day (30–35 dpi). ** The viral replication window refers to the period from the first detection of viral replication in the blood until the clearance or slaughter day.

**Table 5:** Genetic variations (marked in red) identified in the Lv17/WB/Rie1-ΔCD vaccine candidate across sequential in vivo passages.

Nt Position in Lv17/WB/Rie1-∆CD	Genome Region	Function	Reference Lv17/WB/Rie1 (OR863253)	Parental Lv17/WB/Rie1-∆CD	P1	P4	P5	Protein Variation/Comment
8399	NCR	non-coding region	G	G	G	G	A	Intergenic region between MGF-110-2L/3L
15,880	MGF 110-*14L*	unknown (not involved in virulence)	9C	11C	10C	9C/10C	10C	Potential frameshift. Possible error in large homopolymer region
21,364	MGF 300-*1L*	potential role in viral replication in vitro and in vivo	C	C	C	C	T	Synonymous mutation → no aa change
21,450			C	C	C	C	T	A88T

## Data Availability

The data presented in this study are available in this article and Supplementary Materials.

## Supplementary Materials

The following supporting information can be downloaded at https://www.mdpi.com/article/10.3390/vaccines12121406/s1, Table S1: Primers used for the creation of recombinant transfer plasmids and sgRNAs. Table S2: ASFV detection in tissues determined by real-time PCR in domestic pigs immunized either with the marker vaccines Lv17/WB/Rie1-ΔEP153R (M1 to M6) or Lv17/WB/Rie1-ΔUK (M7 to M12) or with the parental strain Lv17/WB/Rie1 (M13 to M18). Table S3: ASFV detection in tissues determined by real-time PCR in domestic pigs immunized with Lv17/WB/Rie1-ΔCD/ΔUK (Group 2.1), Lv17/WB/Rie1-ΔCD (Group 2.2), or the parental strain Lv17/WB/Rie1 (Group 2.3). Table S4: Genome variants found in each passage after in vivo assays using the CLC Genomics Workbench.
