# Supplementary material for: Double Deletion of EP402R and EP153R in the Attenuated Lv17/WB/Rie1 African Swine Fever Virus (ASFV) Enhances Safety, Provides DIVA Compatibility, and Confers Complete Protection Against a Genotype II Virulent Strain
Source: Vaccines (Basel). 2024 Dec 13;12(12):1406. doi: 10.3390/vaccines12121406 (PMC11680264; doi:10.3390/vaccines12121406)
Supplement: Supplementary file 1 [file vaccines-12-01406-s001.zip › Supplementary Table S1.pdf]

Supplementary table S1. Primers used for the creation of recombinant transfer plasmids and sgRNAs

A, Lv17/WB/Rie1-ΔEP153R, transgene: eGFP

Transfer plasmid		
Name of primers	Seuqences	Name and length of PCR fragments
EP153_leftarm_F	GGCCAGTGAATTCGAGCTCGGTACGCTTAATTTAACAGATGCAATC	left homology arm, 1030 bp
EP153_leftarm_p72_R	TATAATGTTATAAAAATAATTTATTGTTTTTATTAAATATTAATATACTATAATCATCTATTT	
EP153_GFP_p72_F	TATTTAATAAAAACAATAAATTATTTTTATAACATTATATATGGTGAGCAAGGGCGAGGAG	eGFP, 776 bp
EP153_GFP_R	TTTTTTGTAATATCCACTATCTTACTTGTACAGCTCGTCCATG	
EP153_rightARM_F	GCATGGACGAGCTGTACAAGTAAGATAGTGGATATTACAAAAAACAAA	right homology arm, 1039 bp
EP153_rightARM_R	TGACCATGATTACGCCAAGCTTGCATGCCACGGTTTAGGTAAGGGAAATG	

Protospacers for sgRNA	
CrispEP153p72-F	CACCGGATTATAGTATATTAATAAT
CrispEP153p72-R	AAACATTATTAATATACTATAATCC
Crisp153END-F	CACCGTCAGTACTATTACGTGATAG
CrispE153END_R	AAACCTATCACGTAATAGTACTGAC

B, Lv17/WB/Rie1-ΔCD, transgene: eGFP

Transfer plasmid		
Name of primer	Sequences	Name and length of PCR fragments
CD_leftarm_F	CGGCCAGTGAATTCGAGCTCGGTACGTTGCTTAATTTAACAGATGCAAT	left homology arm, 969 bp
CD_leftarmP72_R	ATATAATGTTATAAAAATAATTTATTGTTTTTATTAAATATACTTTTTGTTAGAAAACATG	
CD_p72_GFP_F	TATTTAATAAAAACAATAAATTATTTTTATAACATTATATATGGTGAGCAAGGGCGAGGAG	eGFP, 776 bp
CD_GFP_R	GTGAATAAGCGAAATATTACTTGTACAGCTCGTCCATGCCGAGAGT	
CD_rightarm_F	GGCATGGACGAGCTGTACAAGTAATATTTCGCTTATTCACGTAGATAG	right homology arm, 989 bp
CD_rightarm_R	CCATGATTACGCCAAGCTTGCATGCCTCGCTTCGGCTCGCTTCATTCCC	

Protospacers for sgRNA	
Crisp_CD_p72_F	CACCGTTTTCTAACAAAAGTACAT

Transfer plasmid		
Name of primer	Sequences	Name and length of PCR fragments
UK_leftarm_F	AGTGAATTCGAGCTCGGTACGGATGTTAGA AATAGTATTGGCAAC	left homology arm, 1008 bp
UK_p72_leftarm_R	ATATAATGTTATAAAAATAATTTATTGTTTT TATTAAATAGGTATATTAAATCTTAAAATTA TG	
UK_p72_dsRED_F	TATTTAATAAAAACAATAAATTATTTTTATA ACATTATATATGGACAACACCGAGGACG	dsRED, 678 bp
UK_dsred_R	ACTTGCTAAAAAAAATATTTTTTCTACTGGG AGCCGGAGTGGCGGGC	
UK_rightarm_F	CACTCCGGCTCCCAGTAGAAAAAATATTTTT TTTAGCAAGTTTTTAAAC	right homology arm, 1021 bp
UK_rightarm_R	TACGCCAAGCTTGCATGCCACTGGTGGTATC ATCTAATGTTTTG	
Crisp_CD_p72_R	AAACATGTACTTTTTGTTAGAAAAC	
Crisp_CD_END_F	CACCGGTGAATAAGCGAAATATTTT	
Crisp_CD_END_R	AAACAAAATATTTCGCTTATTCACC	

C, Lv17/WB/Rie1-ΔUK, transgene: dsRED- monomeric derivative

Protospacers for sgRNA	
CrispDP96R-F(p72)	CACCGTACAAAAATAGTTTAAGTTT
CrispDP96R-R(p72)	AAACAAACTTAAACTATTTTTGTAC
CrispDP96R-F(end)	CACCGTTTTTAATTATTCTTCTGGA
CrispDP96R-R(end)	AAACTCCAGAAGAATAATTAAAAAC
