# Supplementary material for: Double Deletion of EP402R and EP153R in the Attenuated Lv17/WB/Rie1 African Swine Fever Virus (ASFV) Enhances Safety, Provides DIVA Compatibility, and Confers Complete Protection Against a Genotype II Virulent Strain
Source: Vaccines (Basel). 2024 Dec 13;12(12):1406. doi: 10.3390/vaccines12121406 (PMC11680264; doi:10.3390/vaccines12121406)
Supplement: Supplementary file 1 [file vaccines-12-01406-s001.zip › Supplementary Table S2.pdf]

Table Supplementary S2: ASFV detection in tissues determined by real-time PCR in the domestic pigs either immunized with the marker vaccines Lv17/WB/Rie1-ΔEP153R (M1 to M6) or Lv17/WB/Rie1-ΔUK (M7 to M12) or with the parental strain Lv17/WB/Rie1 (M13 to M18). In grey it is indicated the positive virus isolation (VI) results after three passages in PBM cells. In red is indicated the positive result in Haemadsorption (HAD) indicating the presence of Armenia07 challenged ASFV.

	**Lv17/WB/Rie1-ΔEP153R (GROUP 1.1)**						**Lv17/WB/Rie1-ΔUK (GROUP 1.2)**						**Lv17/WB/Rie1 (parental)**					
**ID domestic pig**	M1	M2	M3	M4	M5	M6	M7	M8	M9	M10	M11	M12	M13	M14	M15	M16	M17	M18
**DPI (DPC)**	19	64 (29)	64 (29)	41 (6)	19	64 (29)	14	13	14	16	13	16	31	30	34	35	35	34
**Liver**	**32.71**	No Ct	No Ct	**34.41**	**26.77**	No Ct	**27.56**	**26.46**	**27.81**	**33.25**	**25.87**	**33.63**	**31.24**	**27.02**	**33.31**	**35.47**	No Ct	**27.3**
**Lung**	**32.41**	No Ct	No Ct	**30.13**	**27.86**	No Ct	**17.31**	**26.29**	**27.38**	**24.84**	**26.5**	**36.95**	**26.86**	**23.44**	**28.85**	**23.15**	**37.51**	**22.1**
**Kidney**	**33.64**	No Ct	No Ct	**36.67**	**30.27**	No Ct	**28.77**	**27.11**	**29.52**	**33.27**	**27.31**	**35.64**	**32**	**27.9**	**32.2**	**35.45**	No Ct	**29.63**
**Heart**	**33.42**	**39.98**	**38.31**	**30.57**	**32.33**	No Ct	**25.71**	**30.99**	**24.63**	**36.79**	**27.1**	**34.88**	**33.51**	**26**	**30.31**	**28.53**	**36.33**	**27.05**
**Spleen**	**29.2**	No Ct	No Ct	**33.24**	**25.13**	No Ct	**29.87**	**26.38**	**26.45**	**33.53**	**24.44**	**35.42**	**31.02**	**28.81**	**31.51**	**31.29**	**38.35**	**27.55**
**Tonsil**	**29.96**	No Ct	No Ct	**32.76**	**29.70**	**31.19**	**27.51**	**27.03**	**28.59**	**26.8**	**25.53**	No Ct	**31.75**	**27.45**	**28.33**	**26.25**	**37.56**	**26.67**
**Renal LN***	**31.05**	**34.85**	No Ct	**32.43**	**28.83**	36.17	**23.63**	**25.24**	**29.41**	**30.93**	**24.87**	No Ct	**25.88**	**24.59**	**30.48**	**31.91**	No Ct	**27.43**
**Retropharyngeal LN**	**30.97**	**39.44**	No Ct	**35.72**	**25.02**	**29.10**	**27.17**	**19.35**	**25.22**	**28.03**	**24.2**	No Ct	**25.48**	**28.15**	**28.66**	**31.95**	**33.02**	**22.84**
**Gastro-hepatic LN**	**33.17**	**34.17**	No Ct	**32.75**	**30.80**	No Ct	**30.66**	**25.98**	**27.54**	**31.92**	**28.22**	**35.81**	**25.84**	**29.8**	**29.88**	**32.3**	No Ct	**31.92**
**Mesenteric LN**	**32.37**	**39.52**	**35.05**	**36.98**	**30.54**	**34.49**	**30.62**	**28.4**	**29.93**	**34.52**	**22.56**	**38.5**	**32.05**	**29.86**	**33.91**	**34.92**	No Ct	**30.83**
**Mediastinal LN**	**30.3**	No Ct	**35.17**	**35.12**	**27.23**	**28.44**	**25.28**	**25.45**	**28.75**	**30.11**	**23.8**	**33**	**27.01**	**22.91**	**31.62**	**30.91**	**33.28**	**26.34**
**Inguinal LN**	**26.4**	No Ct	No Ct	**32.36**	**24.11**	**31.35**	**26.57**	**23.73**	**21.77**	**25.32**	**21.45**	**35.87**	**24.87**	**24.37**	**28.89**	**31.08**	**32.8**	**25.21**
**Submandibular LN**	**27.45**	No Ct	No Ct	**36.69**	**23.22**	**31.63**	**26.53**	**25.32**	**22.35**	**25.52**	**24.89**	**37.75**	**26.22**	**22.96**	**29.04**	**29.41**	**36.9**	**26.56**
**Splenic LN**	**32.72**	No Ct	No Ct	ns	**32.45**	**35.45**	ns	**29.65**	**30.07**	**34.82**	**28.71**	ns	**30.18**	**29.69**	**33.46**	**30.34**	**36.68**	**28.91**
**Popliteal LN**	**23.98**	**39.02**	**38.90**	**19.44**	**22.14**	**29.98**	**25.39**	**22.28**	**24**	ns	**17.9**	**35.46**	**26.12**	**21.55**	ns	**27.55**	**31.35**	**20.55**
**Bone marrow**	**33.54**	No Ct	No Ct	**36.05**	**27.59**	No Ct	**28.01**	**26.25**	**29.21**	**35.34**	**21.81**	No Ct	**32.81**	**26.47**	**32.44**	**33.72**	No Ct	**26.42**
**Diaphragm**	**33.57**	No Ct	**38.72**	**31.01**	**31.76**	No Ct	**27.24**	**27.86**	**29.88**	**30.6**	**29.48**	**33.59**	**28.61**	**26.01**	**33.88**	**30.55**	No Ct	**27.69**
**Front left IA****	**25.75**	No Ct	No Ct	**29.71**	**19.12**	**34.87**	**22.76**	**21.81**	**21.23**	**22.12**	**18.71**	**33**	**23.92**	**23.43**	**23.11**	**25.89**	**31.6**	**19.16**
**Front right IA**	**25.66**	No Ct	**35.72**	**34.44**	**18.84**	**38.72**	**21.38**	**19.37**	**20.79**	**23.65**	**20.12**	**37.14**	**22.43**	**21.47**	**23.29**	**28.88**	**32.41**	**18.86**
**Back left IA**	**25.1**	**39.75**	No Ct	**34.40**	**22.28**	No Ct	**16.57**	**22.03**	**19.47**	**23.73**	**19.28**	**33.31**	**21.2**	**20.32**	**28.78**	**23.53**	**32.81**	**19.58**
**Back right IA**	**25.16**	No Ct	No Ct	**29.47**	**21.61**	**38.34**	**20.13**	**22.31**	**21.47**	**25.08**	**17.95**	**36.62**	**20.44**	**19.11**	**19.79**	**25.65**	**33.96**	**20.04**
**TOTAL PCR POS.**	21/21 (100%)	07/21 (33.3%)	06/21 (28.6%)	20/20 (100%)	21/21 (100%)	11/21 (52.3%)	20/20 (100%)	21/21 (100%)	21/21 (100%)	20/20 (100%)	21/21 (100%)	16/20 (80%)	21/21 (100%)	21/21 (100%)	20/20 (100%)	19/21 (90.7%)	14/21 (58.3%)	21/21 (100%)
**TOTAL VI POS.**	21/21 (100%)	0/21 (0%)	0/21 (0%)	07/20 (35%)	21/21 (100%)	05/21 (23.8%)	20/20 (100%)	21/21 (100%)	21/21 (100%)	18/20 (90%)	21/21 (100%)	05/20 (25%)	21/21 (100%)	21/21 (100%)	20/20 (100%)	19/21 (28.6%)	7/21 (33.3%)	21/21 (100%)

*Lymph node; ** articular cartilage; ns = not sample;
