# Supplementary material for: Double Deletion of EP402R and EP153R in the Attenuated Lv17/WB/Rie1 African Swine Fever Virus (ASFV) Enhances Safety, Provides DIVA Compatibility, and Confers Complete Protection Against a Genotype II Virulent Strain
Source: Vaccines (Basel). 2024 Dec 13;12(12):1406. doi: 10.3390/vaccines12121406 (PMC11680264; doi:10.3390/vaccines12121406)
Supplement: Supplementary file 1 [file vaccines-12-01406-s001.zip › Supplementary Table S3.pdf]

Supplementary Table S3. ASFV detection in tissues determined by real-time PCR in domestic pigs immunized with Lv17/WB/Rie1-ΔCD/ΔUK (Group 2.1), Lv17/WB/Rie1-ΔCD (Group 2.2), or the parental strain Lv17/WB/Rie1 (Group 2.3). Grey cells indicate positive virus isolation (VI) results after three passages in porcine blood monocytes (PBM) without haemadsorption (HAD). Red cells indicate positive HAD results, denoting the presence of Arm07 ASFV in the immunized groups.

**ID domestic pig (DPI/DPC) /Tissue**	Group 2.1 (Lv17/WB/Rie1-ΔCD/ΔUK)					Group 2.2 (Lv17/WB/Rie1-ΔCD)					Group 2.3 (Lv17/WB/Rie1)				
	**C1 (D61/31)**	**C2 (D68/38)**	**C3 (D63/33)**	**C4 (D61/31)**	**C5 (D63/33)**	**C6 (D62/32)**	**C7 (D68/38)**	**C8 (D62/32)**	**C9 (D63/34)**	**C10 (D63/34)**	**C16 (D19)**	**C17 (D62/32)**	**C18 (D12)**	**C19 (D63/33)**	**C20 (D64/34)**
Liver	No ct	No ct	No ct	No ct	No ct	No ct	No ct	**37.24**	No ct	No ct	**34.00**	No ct	**29.52**	No ct	No ct
Lung	**38.74**	**38.92**	No ct	No ct	No ct	No ct	**31.35**	**38.27**	**36.55**	No ct	**30.20**	No ct	**24.46**	**39.71**	No ct
Kidney	No ct	No ct	**37.76**	No ct	No ct	No ct	No ct	No ct	No ct	**39.57**	**35.66**	No ct	**32.78**	**39.45**	No ct
Heart	No ct	No ct	**35.03**	No ct	No ct	No ct	No ct	No ct	**38.85**	**39.41**	**33.36**	No ct	**28.81**	**38.76**	No ct
Spleen	No ct	No ct	**34.61**	No ct	No ct	**39.47**	**39.33**	**33.89**	No ct	No ct	**31.44**	No ct	**33.6**	No ct	No ct
Tonsil	No ct	**31.18**	No ct	No ct	**31.21**	**38.48**	**33.27**	**25.84**	**32.33**	**36.44**	**27.88**	**32.54**	**29.6**	No ct	**34.89**
Renal LN*	**38.99**	**28.44**	**37.21**	No ct	**29.50**	**33.97**	**37.09**	**31.28**	No ct	No ct	**30.93**	No ct	**29.13**	**39.15**	No ct
Retropharyngeal LN	**30.39**	**35.49**	**34.86**	No ct	**37.26**	No ct	**36.57**	**38.09**	No ct	No ct	**29.44**	No ct	**27.52**	No ct	No ct
Gastro-hepatic LN	**38.10**	**32.71**	**34.9**	No ct	**31.21**	**36.85**	No ct	**34.60**	**37.96**	**33.73**	**31.91**	**38.51**	**28.02**	**38.40**	No ct
Mesenteric LN	No ct	**38.90**	No ct	No ct	No ct	No ct	No ct	**32.22**	No ct	No ct	**32.64**	No ct	**29.14**	**39.44**	No ct
Mediastinal LN	**34.59**	**35.21**	No ct	No ct	**34.08**	No ct	**35.30**	No ct	**37.71**	**37.68**	**27.98**	**35.06**	**29.66**	**36.54**	**39.18**
Inguinal LN	No ct	**37.72**	**37.96**	No ct	**31.01**	No ct	**38.08**	**39.22**	No ct	**34.15**	**22.68**	No ct	**24.33**	No ct	No ct
Submandibular LN	**34.02**	**34.96**	**35.86**	No ct	**31.13**	**39.58**	**37.99**	**34.51**	**35.19**	No ct	**26.46**	**36.77**	**25.98**	**34.71**	**38.35**
Splenic LN	No ct	**35.74**	**34.31**	**37.70**	No ct	**37.41**	No ct	**38.14**	No ct	No ct	**32.60**	No ct	**29.77**	No ct	No ct
Popliteal LN	**38.37**	**34.50**	**34.50**	No ct	No ct	No ct	No ct	NT	37.15	**38.20**	**24.37**	**37.80**	**24.83**	No ct	**36.68**
Bone marrow	**39.44**	No ct	No ct	No ct	No ct	No ct	**37.22**	No ct	No ct	No ct	**33.23**	No ct	**30.6**	No ct	No ct
Diaphragm	No ct	No ct	No ct	No ct	No ct	**39.21**	No ct	**39.26**	No ct	No ct	**31.14**	No ct	**30.98**	No ct	No ct
Front left IA**	No ct	No ct	No ct	No ct	No ct	No ct	No ct	No ct	No ct	No ct	**22.37**	**39.31**	**22.5**	**36.90**	**37.07**
Front right IA	No ct	**38.09**	No ct	No ct	No ct	No ct	No ct	**35.71**	38.87	No ct	**22.98**	No ct	**21.04**	No ct	36.94
Back left IA	**38.36**	No ct	No ct	**29.22**	No ct	No ct	No ct	**38.35**	No ct	No ct	**20.83**	No ct	**19.56**	No ct	No ct
Back right IA	**32.06**	**33.26**	No ct	**38.42**	No ct	No ct	No ct	No ct	No ct	No ct	**20.63**	No ct	**20.78**	**36.63**	**38.68**
**TOTAL PCR POS.**	10/21 (47.6%)	13/21 (61.9%)	10/21 (47.6%)	3/21 (14.3%)	7/21 (33.3%)	7/21 (33.3%	9/21 (42.9%)	14/20 (70%)	8/21 (38.1%)	7/21 (33.3%)	21/21 (100%)	6/21 (28.6%)	21/21 (100%)	10/21 (47.6%)	7/21 (33.3%)
**TOTAL VI POS**	3/21 (14.8%)	1/21 (4.7%)	1/21 (4.7%)	1/21 (4.7%)	3/21 (14.8%)	0/21 (0%)	0/21 (0%)	3/21 (14.8%)	1/21 (4.7%)	2/21 (9.5%)	17/21 (80.9%)	0/21 (0%)	21/21 (100%)	0/21 (0%)	0/21 (0%)
